# Improved emotion regulation after neurofeedback: A single-arm trial in patients with borderline personality disorder

**DOI:** 10.1016/j.nicl.2019.102032

**Published:** 2019-10-16

**Authors:** Jenny Zaehringer, Gabriele Ende, Philip Santangelo, Nikolaus Kleindienst, Matthias Ruf, Katja Bertsch, Martin Bohus, Christian Schmahl, Christian Paret

**Affiliations:** a Department of Psychosomatic Medicine and Psychotherapy, Central Institute of Mental Health, Medical Faculty Mannheim/University of Heidelberg, Mannheim, Germany; b Department Neuroimaging, Central Institute of Mental Health, Medical Faculty Mannheim / University of Heidelberg, Mannheim, Germany; c Chair of Applied Psychology/Mental Health Lab, Institute of Sport and Sports Science, Karlsruhe Institute of Technology, Karlsruhe, Germany; d Institute of Psychiatric and Psychosomatic Psychotherapy, Central Institute of Mental Health, Medical Faculty Mannheim/University of Heidelberg, Mannheim, Germany; e Department for General Psychiatry, Center of Psychosocial Medicine, University of Heidelberg, Heidelberg, Germany; f Sagol Brain Institute, Wohl Institute for Advanced Imaging, Tel-Aviv Sourasky Medical Center and School of Psychological Sciences, Tel-Aviv University, Tel-Aviv, Israel; g Department of Psychology, LMU Munich, Munich, Germany

**Keywords:** Borderline personality disorder, Amygdala, Real-time fMRI, Neurofeedback, Emotion regulation

## Abstract

Real-time functional magnetic resonance imaging (fMRI) neurofeedback training of amygdala hemodynamic activity directly targets a neurobiological mechanism, which contributes to emotion regulation problems in borderline personality disorder (BPD). However, it remains unknown which outcome measures can assess changes in emotion regulation and affective instability, associated with amygdala downregulation in a clinical trial. The current study directly addresses this question. Twenty-four female patients with a DSM-IV BPD diagnosis underwent four runs of amygdala neurofeedback. Before and after the training, as well as at a six-weeks follow-up assessment, participants completed measures of emotion dysregulation and affective instability at diverse levels of analysis (verbal report, clinical interview, ecological momentary assessment, emotion-modulated startle, heart rate variability, and fMRI). Participants were able to downregulate their amygdala blood oxygen-dependent (BOLD) response with neurofeedback. There was a decrease of BPD symptoms as assessed with the Zanarini rating scale for BPD (ZAN-BPD) and a decrease in emotion-modulated startle to negative pictures after training. Further explorative analyses suggest that patients indicated less affective instability, as seen by lower hour-to-hour variability in negative affect and inner tension in daily life. If replicated by an independent study, our results imply changes in emotion regulation and affective instability for several systems levels, including behavior and verbal report. Conclusions are limited due to the lack of a control group. A randomized controlled trial (RCT) will be needed to confirm effectiveness of the training.

## Highlights

• To date it has been unclear which aspects of psychopathology and emotion regulation may change with neurofeedback-aided amygdala down-regulation. The present study provides an empirical basis for informed decision-making on primary outcome measures for larger clinical trials of amygdala neurofeedback training.
• Participants were able to down-regulate their amygdala BOLD response with neurofeedback.
• After neurofeedback training, there was a decrease of borderline symptoms as assessed with the Zanarini rating Scale for Borderline Personality Disorder (ZAN-BPD).
• Patients also indicated less affective instability, indicated by lower variability in negative affect and inner tension in daily life after training.
• After training, patients showed decreased emotion-modulated startle to negative pictures, indicative of increased emotion regulation abilities.

## 1 Introduction

Emotion dysregulation is considered a hallmark of borderline personality disorder (BPD) (Glenn and Klonsky, 2009; Sanislow et al., 2002; Schmahl et al., 2014), characterized by heightened reactivity to negative stimuli, with impairments in the implementation and maintenance of adaptive and appropriate emotion regulation strategies, as well as heightened experience of negative affect (Carpenter and Trull, 2013). On a neural level, a key feature of BPD is hyperactivation of the amygdala in response to negative and neutral stimuli (Schulze et al., 2019), likely reflecting the emotion dysregulation observed in BPD patients (Schmahl et al., 2014).

Current emotion regulation models implicate downregulation of the amygdala as a mechanism to control emotions in clinical contexts (Buhle et al., 2014; Etkin et al., 2015). A normalization of amygdala activation and improved emotion regulation were found during Dialectical Behavior Therapy (DBT) in BPD patients, suggesting that amygdala response is an important indicator of BPD remission (Goodman et al., 2014; Schmitt et al., 2016). However, it is not clear whether decreased amygdala response mediates BPD remission. Until recently, probing this has been virtually impossible, as techniques to tackle subcortical activation were limited to highly invasive deep-brain stimulation.

With the emergence of real-time functional magnetic resonance imaging (fMRI), modulation of emotion brain circuitry became feasible (Linhartová et al., 2019). With feedback from brain activation in real-time, dubbed neurofeedback, healthy subjects (Brühl et al., 2014; Herwig et al., 2019; Keynan et al., 2016; Paret et al., 2014) and patients (Nicholson et al., 2017; Paret et al., 2016) were able to reduce their amygdala activation during real-time fMRI. The benefits of this new technique are two-fold: first, assessing behavioral sequels of neuromodulation provide a better understanding of mechanisms that contribute to reduced amygdala activation in BPD. Second, the potential to address dysregulated neurobiological mechanisms with neurofeedback could be used for BPD treatment. However, before addressing these goals, primary outcome measures for clinical trials must be identified.

Emotion dysregulation in BPD has been studied with a plethora of measures, such as emotional picture-viewing tasks (Krause-Utz et al., 2012), clinical interviews (Zanarini et al., 2003), retrospective questionnaires (Glenn and Klonsky, 2009; Gratz et al., 2006; Salsman and Linehan, 2012) and affective variability in ecological momentary assessment (EMA) (Ebner-Priemer et al., 2007; Nica and Links, 2009; Santangelo et al., 2017). In addition, psychophysiological indices such as resting heart rate variability (HRV) and startle modulation have been used to study emotion dysregulation in BPD (Ebner-Priemer et al., 2005; Thompson et al., 2018). BPD patients show lower resting HRV than controls (Koenig et al., 2016), which is indicative of less regulation ability (Appelhans and Luecken, 2010). Cognitive emotion regulation diminishes emotion-modulated startle in healthy individuals (Jackson et al., 2000; Zaehringer et al., 2018) and BPD patients (Thompson et al., 2018), as this downregulation correlates with downregulation of affective states (Zaehringer et al., 2018). Similarly, studies report associations of amygdala hyperactivation and BPD diagnosis (Schulze et al., 2019), outlining a pathway of amygdala regulation via self-injury (Reitz et al., 2015), and reporting a coincidence of amygdala normalization with response to psychotherapy (Goodman et al., 2014). Yet, little action has been shown to map amygdala hyperactivation with behavioral correlates of emotion dysregulation and affective instability. It is unknown what aspects of BPD symptomatology improve with normalization of amygdala activation. Thus, evidence is very limited, impeding informed selection of a primary outcome measure for clinical trials that assess amygdala neuromodulation. The present study addressed exactly this question, i.e., what aspects of emotion dysregulation improve following amygdala neurofeedback? Moreover, because dysfunction of emotion neurocircuitry manifests through dysregulated behavior, including the verbal report of symptoms collected in standard psychometric assessments (LeDoux and Pine, 2016), we used a multimodal assessment of psychopathology, explained below. BPD patients underwent four sessions of neurofeedback training and received a test battery directly before training, both after training and at 6-weeks follow-up. The test battery included a multimodal assessment of emotion regulation of self-report, EMA, behavioral, and fMRI measures. We hypothesized that BPD patients would downregulate the amygdala with neurofeedback. In addition, we hypothesized that BPD patients would show significant changes at several system levels, i.e., verbal report, everyday experience, and behavior and brain responses. Specifically, we hypothesized reductions in emotion dysregulation and improved clinical symptoms, enhanced emotion regulation as shown by increased resting HRV, improved emotion regulation in an established laboratory task (Jackson et al., 2000), and decreased amygdala response to emotional pictures. In addition, we explored changes in everyday experience as well as changes in a number of aspects of emotion regulation and BPD psychopathology.

## 2 Methods and materials

### 2.1 Participants

Twenty-six female patients with at least 5 BPD criteria, according to the DSM-IV (American Psychiatric Association, 2000) participated in the present study. All participants were on stable medication (see Table 1 for details on medication) during the course of the study. In case participants receiving psychotherapeutic treatment, they were required to maintain it throughout the study. Two patients were excluded after completion (one patient reported amphetamine consumption during participation, and one patient fell asleep during the neurofeedback runs).

**Table 1:** Means (SD) of demographics, clinical characteristics and questionnaire data

	T0		T1		T2		*Test-Statistics*			
							*F*	*df*	*p*	
*Demographics*										
Age mean (SD)	33.42	(11.10)								
*Clinical Characteristics*										
Number of BPD criteria fulfilled (DSM-V)	6.61	(1.03)								
*Borderline Symptoms ZAN-BPD*										
Total (SD)	7.48	(3.88)	5.09	(3.99)	5.55	(3.33)	5.13	2, 46	.01	T0 > T1
Affect (SD)	2.9	(1.36)	2.09	(1.41)	2.30	(1.25)	3.43	2, 46	.04	T0 > T1
Cognition (SD)	2.17	(1.74)	1.35	(1.46)	1.25	(1.17)	4.52	2, 46	.02	T1 > T2
Impulsivity (SD)	0.96	(1.46)	0.96	(1.43)	0.85	(0.80)	0.11	1.57, 36.14	.85	
Interpersonal Relationships (SD)	1.48	(1.13)	0.96	(1.30)	1.15	(1.51)	3.82	2, 46	.03	T0 > T1
*Difficulties with Emotion Regulation Scale (DERS)*										
Total (SD)	123.29	(17.49)	121.71	(18.73)	113.20	(20.88)	3.80	2, 46	.03	
Nonaccepatence (SD)	20.79	(5.32)	21.62	(5.56)	19.45	(4.33)	2.08	2, 46	.14	
Goals (SD)	20.08	(3.37)	19.25	(3.76)	18.44	(3.72)	3.70	2, 46	.03	T0 > T2
Impulse (SD)	18.21	(4.01)	17.63	(3.77)	16.96	(4.18)	1.02	2, 46	.37	
Awareness (SD)	20.21	(5.30)	19.75	(5.75)	18.79	(5.38)	3.18	2, 46	.05	
Strategies (SD)	28.79	(4.73)	28.58	(.,52)	25.86	(5.98)	4.52	2, 46	.02	T0 > T2,T1 > T2
Clarity (SD)	15.21	(4.18)	14.88	(3.95)	13.69	(3.76)	1.99	2, 46	.25	
*Affect Lability Scale (ALS)*										
Total (SD)	89.42	(16.86)	92.89	(17.56)	88.98	(18.66)	0,45	2, 46	.64	
Depression	17.33	(4.53)	18.76	(4.31)	19.18	(3.88)	1.37	2, 46	.26	
Elation	20.04	(5.69)	18.98	(6.11)	18.65	(6.68)	.48	2, 46	.62	
Depression Elation	13.88	(4.06)	14.51	(4.51)	14.89	(4.34)	.45	2, 46	.67	
Anxiety	12.17	(2.53)	13.90	(3.51)	12.25	(3.62)	2.41	2, 46	.10	
Anger	12.63	(2.60)	12.10	(3.26)	10.89	(3.43)	2.28	2, 46	.11	
Anxiety Depression	13.38	(3.66)	14.59	(4.08)	13.12	(4.40)	0.90	2, 46	.40	
*Toronto Alexithymia Scale (TAS-26)*										
Total (SD)	54.17	(10.29)	53.50	(9.56)	50.54	(10.78)	2.76	1.36, 31.22	.096	
Identification of one's feelings	22.67	(4.05)	21.29	(3.98)	19.82	(4.84)	6.25	2, 46	.004	T0 > T2
Difficulty Describing Feelings	15.96	(3.61)	16.58	(3.93)	15.39	(3.83)	1.39	2, 46	.26	
External thinking	15.54	(4.81)	15.63	(4.99)	15.33	(4.97)	0.05	1.47, 33.72	.95	
*Emotion Regulation Skills Questionnaire*										
Total (SD)	78.61	(21.31)	77.87	(20.27)	86.83	(18.26)	5.90	2, 46	.01	T0 < T2
Awareness	9	(3.24)	9.71	(3.45)	10.27	(2.90)	3.85	2, 46	.03	T1 < T2
Clarity	9	(2.47)	9.00	(2.96)	9.63	(2.49)	4.65	10.31, 2.22	.03	T0 < T2
Sensations	8.58	(2.98)	8.25	(2.94)	10.33	(2.63)	4.08	2, 46	.02	T0 < T2
Understanding	9.33	(3.23)	8.71	(3.16)	9.73	(2.43)	4.64	2, 46	.02	T1 < T2
Accepatnce	8.5	(2.87)	8.37	(2.39)	8.59	(2.76)	5.96	2, 46	.01	T1 < T2
Tolerance	8.06	(2.86)	7.83	(2.55)	9.77	(2.86)	0.90	2, 46	.41	T1 < T2
Readiness to confront distressing situations	9.58	(3.12)	9.25	(3.30)	9.45	(2.71)	0.17	2, 46	.84	
Self-support	8.75	(2.79)	8.79	(2.70)	9.77	(2.85)	3.62	2, 46	.04	
Modification	7.79	(2.77)	7.96	(2.74)	8.91	(2.26)	2.88	2, 46	.07	
*UPPS*										
Urgency (SD)	2.9	(0.46)	–	–	–	–				
Pre (SD)	2.37	(0.45)	–	–	–	–				
Pers (SD)	2.54	(0.51)	–	–	–	–				
SS (SD)	2.76	(0.54)	–	–	–	–				
*DSS-21*										
Intensity (SD)	18.49	(11.65)	14.80	(9.42)	14.48	(10.43)	2.16	1.36, 31.32	.12	
Duration (SD)	18.49	(1.28)	2.24	(1.42)	2.18	(1.36)	1.47	1.23, 35.41	.24	
*Current comorbidities N (%)*										
Major Depression	6	(24%)								
Major Depression lifetime	22	(88%)								
Dysthymia	4	(16%)								
Double Depression	3	(12%)								
Panic Disorder	3	(12%)								
Social Phobia Disorder	4	(16%)								
Specific Phobia	5	(20%)								
PTBS	6	(24%)								
Anorexia Nervosa	1	(4%)								
Bulimia Nervosa	1	(4%)								
Binge Eating Disorder	2	(8%)								
*Psychotropic Medication N (%)*										
SSRI	3.00	(12.50)								
SNRI	4.00	(16.70)								
Tricyclica	3.00	(12.50)								
Other Antidepressants	3.00	(12.50)								
Neuroleptics	5.00	(20.80)								
Anticonvulsants	2.00	(8.30)								
Unmedicated	10.00	(41.70)								

*Notes:* SD, standard deviation; UPPS, Impulsive Behavior Scale; DSS-21, Dissociation Tension Scale; SSRI, Selective serotonin reuptake inhibitor; SNRI, Serotonin–norepinephrine reuptake inhibitor.

The diagnostic assessment comprised the Structured Interview for DSM-IV Axis-I (First et al., 1997) and the International Personality Disorder Examination (Loranger, 1999). Patients were excluded from our study in cases of severe somatic illness and if exclusion criteria related to MRI were fulfilled (metal implants, left-handedness, claustrophobia, and pregnancy). Further exclusion criteria were alcohol or substance abuse within the last 6 months, lifetime psychotic disorder, bipolar affective disorder, or mental retardation.

A total of *n* = 108 individuals were initially screened for our study. *N* = 77 had to be excluded because they did not fulfill our inclusion criteria, were not interested in the first place or were interested but ultimately did not participate. Thus *n* = 31 participants were allocated to our study and *n* = 26 of them received the full neurofeedback training. A detailed flow chart of the study is shown in Fig. S1 in the supplement.

Descriptive statistics of demographic variables are reported in Table 1. The study was approved by the Ethics Committee of the Medical Faculty Mannheim / Heidelberg University and was conducted according to the Declaration of Helsinki. All subjects gave written informed consent prior to participation and received financial compensation (120 Euros). The research protocol was registered on ClinicalTrials.gov (NCT02866110) and the *Deutsches Register für Klinische Studien* (drks.de; DRKS00009363).

### 2.2 Procedure

**Fig. 1:**
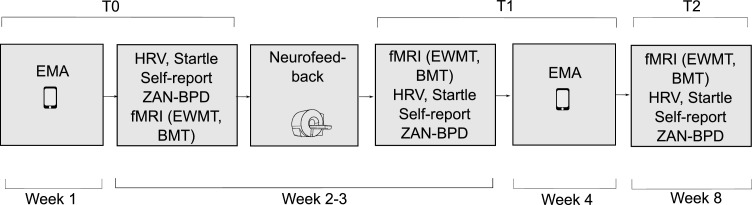
Participants received a total of 4 runs of amygdala neurofeedback training (weeks 2–3). Runs were administered on 3 different days, with run 2 and 3 being administered consecutively on the second training day. At baseline (T0), participants completed an emotion test battery: ecological momentary assessment (EMA) was assessed on 4 consecutive weekdays before neurofeedback started (week 1). At the beginning of week 2, the Zanarini Rating Interview (ZAN-BPD), self-report questionnaires, heart rate variability (HRV), and an emotion regulation task with emotion-modulated startle (Startle) was administered. Participants also answered an Emotional Working Memory Task (EWMT) and a Backward Masking Task (BMT) during fMRI, immediately before completion of the first neurofeedback session. From 2 to 7 days later, participants completed the next neurofeedback session (visit 2), followed by 2–7 days for their third and final session (visit 3). During these sessions, participants were instructed to downregulate a thermometer, with activity of the right amygdala, while watching aversive pictures. Details of the neurofeedback procedure can be found in the supplement. Immediately after the last neurofeedback session (end of week 3), the test battery was administered a second time (T1). The follow-up visit (T2) was completed 6 weeks after visit 3, and was identical to T1, excluding EMA (week 8).

Participants took part in four runs of amygdala neurofeedback training. Runs were administered on 3 different days, with run 2 and 3 being administered consecutively on the second training day. Training days were scheduled 2–7 days apart from each other. At baseline (T0) and after completion of amygdala neurofeedback training (T1), the test battery was administered. All measures except EMA were assessed again at 6-weeks follow-up (T2). For details of the procedure, see Fig. 1. The consensus on the reporting and experimental design of clinical and cognitive-behavioral neurofeedback studies (CRED-nf checklist (Ros et al., 2019, January 23) can be found in the supplement on page 10.

### 2.3 Neurofeedback

#### 2.3.1 Procedure

**Fig. 2:**
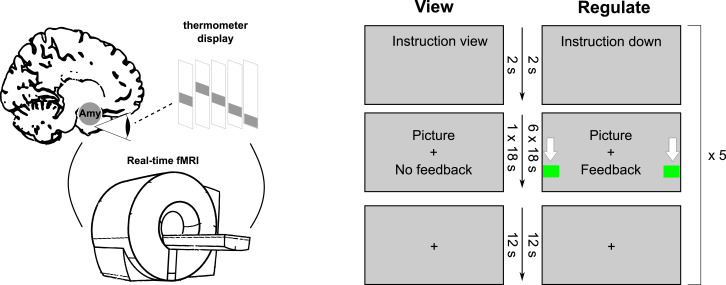
Experimental procedure of a neurofeedback training run. Participants viewed aversive pictures, with a feedback signal from their amygdala BOLD response, which is depicted as a thermometer. They were instructed to try to downregulate the temperature, representing their brain activation. They were also told to consider the temporal delay of the BOLD response, resulting in a time lag of the thermometer response (2–5 s). Furthermore, they should not close their eyes or shift their gaze from the screen and avoid focusing exclusively on the thermometer or borders of the picture. They should not hold their breath or move their heads. After participants entered the scanner, anatomical and fieldmap scans were acquired. Before the first neurofeedback run, a demonstration trial was presented without fMRI scanning. Subjects were instructed beforehand to look at the picture (without feedback), or to downregulate the thermometer signal. The neurofeedback consisted of ‘down’ and ‘view’ conditions, respectively. In the ‘view’ condition, a picture with negative emotional content was shown for 18 s, followed by a fixation cross on a grey background (‘rest,’ 12 s). In the ‘down’ condition, pictures were presented with feedback. Six pictures were presented in a ‘down’ block, each for 18 s (108 s total). The order of conditions was fixed, with alternating ‘view’ and ‘down’ blocks. In total, there were 5 ‘down’ blocks and five ‘view’ blocks. After the last block, participants were instructed to rate their perceived regulation success (‘Were you able to regulate the display?’) on a 10-level visual analogue scale (results can be found in the supplement).

Subjects were instructed to look at negative pictures (without feedback, ‘view’ condition), or downregulate a colored thermometer bar, representing brain activation while watching negative pictures (‘down’ condition), respectively. Participants were not given a particular strategy to downregulate. Rather, they were told to assess what strategy worked best for them. In the ‘view’ condition, a picture with negative emotional content was presented for 18 s, followed by a fixation cross on a grey background (‘rest,’ 12 s). In the ‘down’ condition, pictures were presented with feedback. After each neurofeedback session, participants were asked which strategies they used to downregulate (s. supplement for details). For details, see Fig. 2. Three participants had to be excluded from the statistical analysis due to technical problems in session 2 and 4 (logfiles were not available).

#### 2.3.2 Online fMRI data analysis

The neurofeedback signal was computed as the fMRI percent of signal change, relative to the global mean, and updated every second and displayed as a colored bar. The BOLD signal data were calculated online from voxels within a right amygdala mask, produced with the Harvard-Oxford brain atlas with a probability threshold of 25%. Details of fMRI acquisition, real-time fMRI analysis, and feedback presentation are in the supplement, and were published by Paret et al. (2018).

### 2.4 Assessments

#### 2.4.1 Verbal report: Interviewer- and self-assessment

Self-assessment included several questionnaires on different aspects of BPD psychopathology and emotion regulation.

To test our hypothesis on changes in emotion dysregulation, we used the difficulties with the Emotion Regulation Scale (DERS; Gratz and Roemer, 2004), a 36-item questionnaire that assesses levels of emotion regulation problems. The DERS is comprised of six subscales (nonacceptance of emotional response difficulties in engaging in goal-directed behavior, impulse control difficulties, lack of emotional awareness, limited access to emotion regulation strategies, and lack of emotional clarity). The DERS was found to have adequate construct and predictive validity and good test-retest reliability over a period of 4–8 weeks (ρ_I_ = .88; Gratz and Roemer, 2004).

To test our hypothesis on changes in clinical status, we assessed the Zanarini rating scale for BPD (ZAN-BPD; Zanarini et al., 2003). The ZAN-BPD is a semi-structured interview and reflects a 1-week time frame. The nine criteria for BPD were rated on a five-point anchored rating scale of 0–4 by trained psychologists (JZ, CP, SM), yielding a total score between 0 and 36. The ZAN-BPD demonstrates good reliability (Cronbach's alpha = 0.85), with convergent and discriminant validity (Zanarini et al., 2003). One participant was excluded for the statistical analysis of the ZAN-BPD, because she did not do the interview at T2.

In addition to these two measures, we assessed several other questionnaires which were used for explorative analyses: The German version of the Emotion Regulation Skills Questionnaire (SEK-27; Berking and Znoj, 2008; Grant et al., 2018) was used to assess emotion regulation skills.  The SEK-27 is a 27-item self-report instrument that utilizes a 5-point Likert-type scale (0 = not at all to 4 = almost always) to assess the respondent's adaptive emotion regulation skills the previous week. The SEK-27 comprises six subscales: (1) awareness, (2) clarity, (3) understanding, (4) modification, (5) acceptance, and (6) tolerance. In addition to the subscales, the SEK-27 provides a total score, computed as the average of all items. The SEK-27 showed adequate internal consistency (Cronbach's alpha = .90 for the total score, and .68–.81 for subscales), as well as adequate test-retest reliability (*r* = .75 for total score). The Affective Lability Scale (ALS; Harvey et al., 1989), a 54-item self-report scale, was used to measure changeable affect. ALS items assessed subjects’ perception of their tendency to vary between what they considered a normal mood versus those of anger (ANG), depression (DEP), elation (ELA), and anxiety (ANX), with a tendency to oscillate between depression and elation (BIP), or between states of anxiety and depression (ANXDEP). Each item was rated on a four-point scale (scored 0–3 inclusive) from ‘‘very undescriptive’’ to ‘‘very descriptive’’ of themselves. The ALS total is the mean of six subscales for individual affect shifts, and showed good internal consistency (among subscales, alpha range = .76–.86). The Toronto Alexithymia Scale (TAS-26; Bagby et al., 1994), a 26-item scale, was used to measure alexithymia in three dimensions: *difficulty identifying feelings, difficulty-describing feelings*, and *externally-oriented thinking*. The TAS-26 displays adequate reliability, ranging from *r* = .67 to *r* = .84. We further used the Dissociation Tension Scale (DSS; Stiglmayr et al., 2009) to assess dissociative symptoms, with the short version of the UPPS Impulsive Behavior Scale (Cyders et al., 2014) to control baseline impulsivity.

#### 2.4.2 Everyday experience: EMA

To measure affective instability and emotion regulation during participants’ everyday lives, we used a smartphone programmed with the movisensXS app (Movisens GmbH, Karlsruhe, Germany) as an electronic diary. The e-diary emitted a prompting signal according to a stratified random schedule, with 12 assessments per day between 9:00 a.m. and 10:00 p.m. on four consecutive workdays. Thus, the 13-h assessment period of each day was divided into 12 intervals, with assessments scheduled at random within each one. At each prompt, we assessed participants’ current affective state using five questions about positive affect (PA) and five questions about negative affect (NA), based on the affective circumplex model (Russell, 1980). To assess participants’ current dissociative state, we used the DSS-4, including an item asking about aversive tension (5 items; (Stiglmayr et al., 2009). We also assessed participants’ perceived control over their emotions with two items (“When the phone rang, I felt like I could control my feelings” and **“**When the phone rang, I felt overwhelmed by my feelings”**)**. The wording of all items can be found in the supplement. We determined the person-mean of the repeated assessments, as well as the mean squared for successive differences (MSSD) as an established instability index for each person and for both assessment periods (i.e., for both the pre- (T0) and post-(T1) neurofeedback training EMA assessment).

#### 2.4.3 Behavior and peripheral physiology: Emotion regulation test, resting HRV

**Fig. 3:**
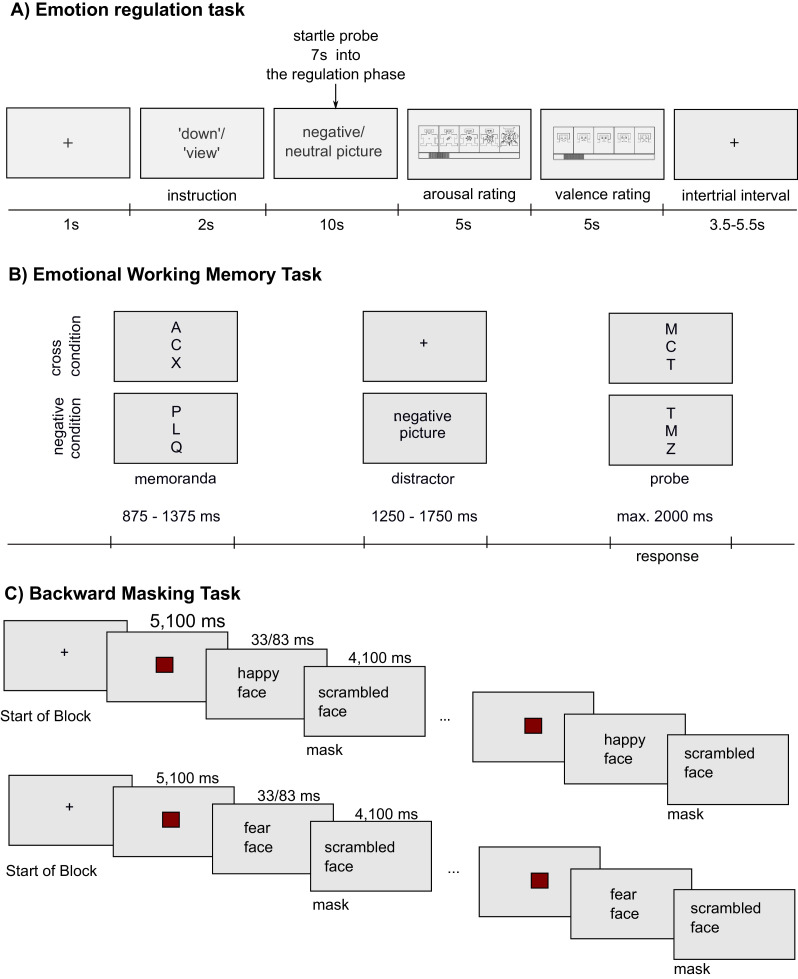
A) Experimental procedure of the emotion regulation task. Participants were instructed either to view negative and neutral pictures without modifying their emotions (‘view’; ‘neutral’ condition, respectively) or to downregulate their feelings toward negative pictures (‘down’ condition). Each trial began with a 2000 ms presentation of an instructional cue (‘view’, ‘down’), followed by a fixation cross displayed for 1000 ms. Next, a neutral or negative picture was presented for 10,000 ms. A startle probe (50 ms, 95 dB white noise burst) was presented through headphones at 6500 ms–9500 ms into the regulation phase). Self-assessment Manikins (SAM Ratings; Bradley and Lang, 1994) were presented after presentation of each picture. Participants rated a 1–9 Likert scale on how positive / negative and aroused / calm they felt at that moment. Lower scores on the valence scale indicated that they felt more positive; lower scores on the arousal scale indicated that they felt calmer. Intertrial intervals were jittered between 3500 and 5500 ms. Details about stimuli and the procedure can be found in the supplement. B) Experimental procedure of the Emotional Working Memory Task (EWMT), which is an adapted Sternberg item recognition task (Sternberg, 1966), modified by Oei and colleagues (Oei et al., 2009; Krause-Utz et al., 2012). Each trial started with the presentation of a set of three letters (memoranda, 875–1375 ms). After a delay phase of 1250–1750 ms, another set of three letters appeared on the screen (probe, 2000 ms). Next, a blank screen appeared (intertrial interval, 550–1050 ms). Participants had to press the left or right button to indicate whether they recognized one of the memoranda-letters in the probe. In half of the trials, one of three memoranda was present. During the delay interval, no distractor (i.e., a fixation cross; ‘cross’ condition) or a distractor (i.e., an aversive picture; ’negative’ condition) was presented. Details of stimuli and the procedure can be found in the supplement. C) Experimental Procedure of the Backward Masking Task (BMT). Participants were instructed to identify photographs of faces expressing happy or fearful facial expressions. The BMT had a total of four conditions: happy or fearful facial expressions presented for 33 ms or 83 ms. A total of 4 blocks per condition were presented. Each block consisted of 8 consecutive pictures. Each block began with a fixation cross. Next, eight faces were shown for 33 ms or 83 ms, preceded by a red rectangle on a grey background for 5100 ms and followed by a mask (scrambled face) for 4100 ms. Details of the stimuli, procedure, and behavioral results are in the supplement.

To test changes in emotion regulation, we assessed the emotion-modulated startle during an emotion regulation paradigm, modified from Jackson et al. (2000). For details of the procedure, see Fig. 3A. In brief, participants were instructed to view negative or neutral pictures (‘view,’ ‘neutral’ condition) or to downregulate emotions in response to negative pictures (‘down’ condition). Seven seconds into the regulation phase, a burst of white noise was presented for 50 ms at 104 dB^1^ (startle probe). The eye blink was measured by electromyogram (EMG). The raw EMG signal was sampled at 1000 Hz, and the gain was amplified by 2000. High-pass (50 Hz) and low-pass (500 Hz) online filters were applied to the data with AcqKnowledge software (version 3.4; BIOPAC Systems; Goleta, CA, USA). EMG data were integrated over 10 samples and analyzed offline with Clip, a C++ based, semi-automated program (Kinzig et al., 2008). Emotion-modulated startle response was defined as the difference between peak (20–120 ms after stimulus onset) and baseline (20 ms prior to stimulus onset) signal. Amplitudes were transformed to T-scores with mean = 50 and SD = 10. Responses were averaged in participants for each condition. Emotion-modulated startle amplitudes in the ‘neutral’ condition were subtracted from the ‘down’ and the ‘view’ conditions for statistical comparison. Four participants (*N* = 4) were excluded for statistical analysis of the startle data due to technical problems at T0 or T1 (*N* = 2). One participant did not show a startle response at all and one participant did not complete the psychophysiological tests at T2.

To test changes in resting HRV, we recorded the electrocardiogram (ECG) for 6 min at 1000 Hz, with a gain of 2000. High-pass (50 Hz) and low-pass (500 Hz) online filters were applied to the data with AcqKnowledge software. Offline, ECG waveforms were transformed into the heart rate (beats per minute) and analyzed with Kubios software (Amsterdam, The Netherlands) (Tarvainen et al., 2014). Resting HRV was calculated as the ratio of low frequency (LF) power in the .04–0.15 Hz range and high frequency (HF) power in the 0.15–0.40 Hz range, indicative of sympathetic to parasympathetic autonomic balance (HF/LF). Three participants (*N* = 3) were excluded: two (*N* = 2) were excluded due to technical problems at T0 or T1, and one (*N* = 1) did not complete the psychophysiological tests at T2.

#### 2.4.4 Brain responses

To test changes in amygdala response during shortly presented pictures, we conducted the EWMT and the BMT in the fMRI. Details of these procedures can be found in Fig. 3B and C. Three participants (*N* = 3) were excluded for the statistical analysis of the EWMT, due to missing button presses all three times (*n* = 2), and technical problems at T1 (*N* = 1; logfile not available). One participant was excluded for the statistical analysis of the BMT due to technical problems at T1 (logfile not available).

### 2.5 Offline fMRI data analysis

#### 2.5.1 Neurofeedback data

##### 2.5.1.1 Preprocessing

Data analysis was performed with Matlab (vR2012a)-based SPM12 package (v6225, Wellcome Trust Center for Neuroimaging, London, UK). Preprocessing included slice timing, which was corrected with reference to the middle slice of a volume, realignment of the scans to the first scan of the series, with rigid body transformation and correction of geometric distortions using a voxel displacement map (VDM); this was produced based on fieldmap scans. The functional scans were not warped, given the VDM parameters and corrected for susceptibility-by-movement artifacts (Andersson et al., 2001). A mean image of the functional scans was next computed and coregistered to the anatomical scan of the subject; this scan was segmented with six standard SPM tissue probability maps and normalized to MNI space. These parameters were used for normalization of functional images. Images were resampled to 2 mm isometric voxels. Functional data were smoothed using an 8 mm kernel (full width at half maximum, FWHM) to account for between-subject variation in anatomical localization. Finally, a high-pass filter (256 s cut-off) was added to the general linear model (GLM) to remove slow signal drifts. An autoregressive model was used to account for serial correlations.

##### 2.5.1.2 Amygdala ROI (Region of Interest) analysis

We estimated HRFs using the inverse logit model by Lindquist et al. (2009) to investigate the hemodynamic amygdala response. First, the eigenvariate was extracted from voxels corresponding to the right amygdala, with the same mask being used for neurofeedback. The eigenvariate was also adjusted for condition effects (‘down’ and ‘view’). HRFs were fitted to each picture presentation interval. The HRF amplitude represents the magnitude of the event-related BOLD response. In addition, we analyzed the area under the curve (AUC). Amplitude estimates and AUC values were compared with SPSS statistics software (v23, IBM Corp. Armonk, NY, USA).

##### 2.5.1.3 Amygdala down-regulation success

We quantified down-regulation success by creating two different indices: First deltas of amygdala amplitudes/AUCs (‘view’ minus ‘down’) between the first and last neurofeedback run were created. However, as this index assumes linear improvement and may misrepresent actual learning slopes, we complemented this by calculating the best performance (Paret et al., 2019) of each participant. That is, we determined the largest delta between the ‘view’ and ‘down’ condition for each participant across all four neurofeedback runs.

#### 2.5.2 EWMT and BMT

##### 2.5.2.1 fMRI data acquisition and analysis

For fMRI acquisition, a 3 Tesla MRI Scanner (Trio, Siemens Medical Solutions, Erlangen, Germany) with a 32-channel head coil was used. T1-weighted anatomical images were acquired with a Magnetization Prepared Rapid Acquisition Gradient Echo sequence (TE = 3.03 ms, TR = 2.3 s, 192 slices and FOV = 256 × 256 mm). Functional images of both EWMT and BMT tasks were acquired with a gradient echo T2* weighted echo-planar-imaging (EPI) sequence with a field of view = 210 mm × 210 mm, voxel size = 3 mm × 3 mm × 3 mm, echo time = 30 ms, TR = 2000 ms with 40 contiguous 3 mm sagittal slices in a 64 × 64 matrix. Head movement artifacts and scanning noise were reduced with head cushions and headphones in the scanner coil. Preprocessing was comprised of adjusting for variable acquisition time over slices (slice-timing), head motion correction (realignment), normalization of images into a standard three-dimensional space defined by the Montreal Neurological Institute (MNI), and spatial smoothing using an 8 mm Gaussian kernel to increase signal-to-noise ratio.

##### 2.5.2.2 First-level analysis

For the EWMT, we modeled regressors for the memoranda, probe and response phase, respectively. In addition, each condition was modeled (negative, cross). Parameter estimates from the contrast of interest (negative > cross) were entered into group-level *t*-tests. For the BMT, we modeled regressors for each condition (happy faces 33 ms, happy faces 83 ms, fearful faces 33 ms, and fearful faces 83 ms). All regressors were convolved with the HRF implemented in SPM12. Parameter estimates from the contrast of interest (all conditions versus implicit baseline) were entered into group-level *t*-tests. To test our hypotheses, voxel-wise *t*-tests of parameter estimates for the EWMT contrast negative > cross, and the BMT contrast (all conditions *versus* implicit baseline) were conducted on the first level. The mean contrast value was then extracted from all voxels of the right amygdala, based on the neurofeedback mask.

### 2.6 Statistical analysis of assessments

Validated statistical software (SPSS v25; IBM Inc., Armonk, NY, USA) was used for analyses. Missing variables were estimated from available items, based on a Stochastic Regression Imputation (SRI) approach, which improves deterministic regression imputation by imputing a value which includes a random error (van Ginkel et al., 2010), hereby avoiding both bias and overfitting (Enders, 2006). For missing self-report items, the regression model underlying SRI was based on all other items from the questionnaire (within the same assessment). For missing neurofeedback, ZAN-BPD, psychophysiological information, EWMT, and BMT variables, the regression model underlying SRI was based on all other conditions available across assessments. We used the stochastic regression imputation SPSS syntax provided by van Ginkel et al. (2010). All variables (including original and imputed data) were entered into repeated-measures: ANOVA with time (T0, T1, and T2) and condition (if available) as within-subject factors (*p < .05). If Mauchly's sphericity test was significant, Greenhouse-Geisser correction was applied to the degrees of freedom.

To limit the risk of false positive results, results from original data are reported in case they differed from results with imputed data. If results from original data do not differ from those with imputed data, the original data without imputation are not reported. We repeated analyses on measures with a-priori hypotheses (i.e. our primary endpoints: ZAN-BPD and DERS total score, emotion-modulated startle, resting HRV and amygdala reactivity to BMT and EWMT) with a conservative correction for multiple tests (i.e. Bonferroni-correction).

In addition, we ran correlation analyses between amygdala down-regulation (deltas of amygdala amplitudes/AUCs subtracting the ‘down’ from the ‘view’ condition) and primary endpoints (i.e. emotion-modulated startle [‘view’ minus ‘down’], resting HRV, amygdala activity to the EWMT [negative > cross contrast] and BMT [all conditions *versus* implicit baseline], ZAN-BPD total score and DERS total score at T0 and T1, respectively). We also ran correlations between down-regulation success indices (deltas of amygdala down-regulation [run 1 minus run 4], best performance) and changes in emotion-modulated startle, resting HRV, amygdala activity to the EWMT and BMT, ZAN-BPD total score and DERS total score using Pearson's r correlation coefficient. Changes in amygdala reactivity during the EWMT (negative > cross contrast) and BMT (all conditions *versus* implicit baseline), emotion-modulated startle (‘view’ minus ‘down’), resting HRV, ZAN-BPD total score and DERS total scores were calculated by subtracting means at T1 from T0. Correlation analyses were limited to our primary endpoints. We did not run correlations with the remaining outcome measures to avoid an increase in chances of false discovery due to multiple testing.

## 3 Results

### 3.1 Amygdala downregulation success

**Fig. 4:**
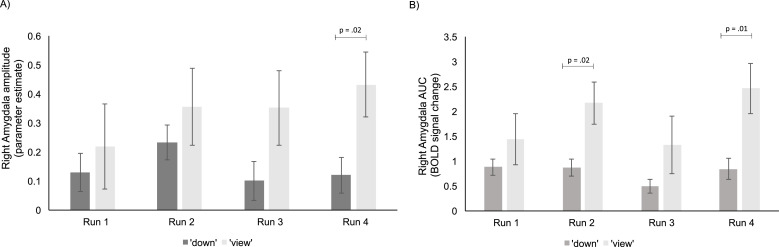
Amygdala amplitude and AUC in the ‘down’ and ‘view’ conditions at each neurofeedback session. A) Participants significantly downregulated the amygdala amplitude with neurofeedback comparing the ‘down’ with the ‘view’ condition at run 4. B) participants significantly downregulated the amygdala AUC at run 2 and run 4. Error bars represent standard error of the mean (SEM). AUC = area under the curve.

Participants downregulated the amygdala BOLD amplitude, *F* (1,23) = 9.40, *p* = .01, eta^2^ = .30. This effect was driven by a significant difference between ‘down’ and ‘view’ at the fourth training run, *t*(23) = −2.51, *p* = .02, *d* = −0.51 - whereas at the first, *t*(23) = -.51, *p* = .61, second, *t*(23) = -.77, *p* = .45, and third, *t*(23) = −1.71, *p* = .10, training run, amygdala BOLD amplitude did not significantly differ between ‘down’ and ‘view’ conditions (see Fig. 4A)). Interaction between condition and run was not significant, *F* (3,69) = .43, *p* = .73, eta^2^ = .02, showing that the observed improvement of amygdala downregulation over time did not pass the significance level.

Statistical analysis did not support the trend for improvement via training. Similar results were seen for the amygdala AUC: participants could downregulate the amygdala AUC, *F* (1,23) = 13.30, *p* < .01, eta^2^ = .37, yet this effect was driven by a significant difference between ‘down’ and ‘view’ at the second, *t*(23) = 2.48, *p* = .02, *d* = .50, and fourth training run, *t*(23) = −2.76, *p* = .01, *d* = −0.56. In the first, *t*(23) = -.97, *p* = .34 and third, *t*(23) = −1.38, *p* = .18 training run, amygdala AUC did not significantly differ between ’down’ and ‘view’ conditions (see Fig. 4B). Interaction between condition and run was not significant, *F* (3,69) = .67, *p* = .57, eta^2^ = .03.

From the amygdala AUC and amplitudes (delta between ‘view’ and ‘down’) we determined the best run (i.e. largest delta) for each participant. When considering the AUC, 6–8 participants each showed best performance during run 2–4, respectively, whereas only two participants showed best performance at the first run (see Fig. S3). When considering the amygdala amplitude, best performance was more equally distributed across runs.

### 3.2 Verbal report

**Fig. 5:**
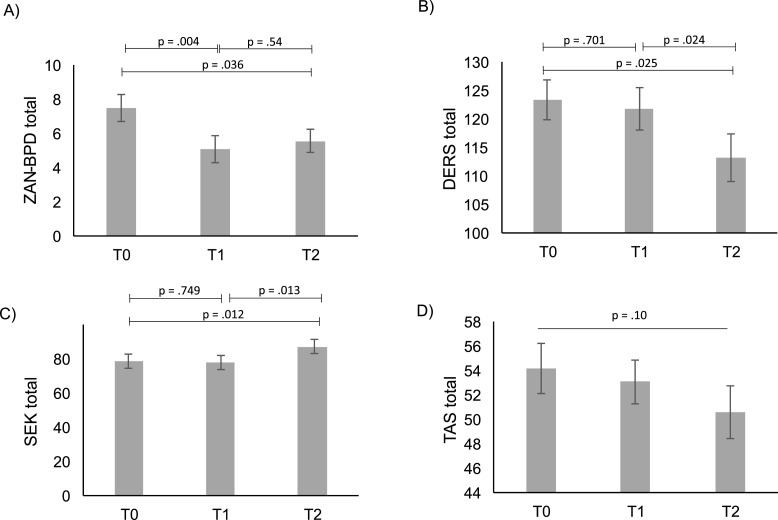
Diagnostic interview and self-assessment results at T0, T1, and T2. A) BPD psychopathology significantly (*p* < .05 uncorrected) improved from T0–T1 and from T0–T2. B) Difficulties with the Emotion regulation Scale (DERS) total score significantly^3^ reduced from T1–T2 and from T0–T2, indicating that difficulties in emotion regulation decreased over time. C) Self-assessment of the emotional competencies (Selbsteinschaetzung Emotionaler Kompetenzen, SEK) total score significantly increased from T1–T2 and from T0–T2, showing an increase in emotional competence over time. D) A trend in reduction of alexithymia was observed, but did not pass the significance test. Error bars represent standard error of the mean (SEM).

The main effect of time of the ZAN-BPD total score revealed that overall BPD symptoms lessened over time, *F*(2, 46) = 5.13, *p* = .010 (uncorrected^2^), eta^2^ = .18. Post hoc paired *t*-tests showed a significant reduction from T0 to T1, *t*(23) = 3.17, *p* = .004, *d* = .65, no significant change from T1 to T2, *t*(23) = -.62, *p* = .54, *d* = -.13, and a significant reduction from T0 to T2, *t*(23) = 2.22, *p* = .036, *d* = .45 (see Fig. 5A). A main effect of time of the DERS total score indicated how difficulties with emotion regulation did change over time, *F*(2,46) = 3.78, *p* = .03 (uncorrected^2^), eta^2^ = .14 (see Fig. 5B). Post hoc paired *t*-tests showed a significant reduction from T1 to T2, *t*(23) = 2.42, *p* = .025, *d* = .49 and from T0 to T2, *t*(23) = −2.40, *p* = .024, *d* = .49. Original data without imputation revealed nonsignificant main effect of time of the DERS total score, *F*(2,40) = 2.48, *p* = .10, eta^2^ = .11.

Explorative analyses showed a significant main effect of time for the SEK total score, *F*(2,46) = 5.90, *p* = .01, eta^2^ = .20, indicating change of emotional competence over time (see Fig. 5C). Post hoc paired *t*-tests revealed that emotion regulation skills and their efficacy significantly increased from T1 to T2, *t*(23) = −2.71, *p* = .01, *d* = -.55, and significantly increased from T0 to T2, *t*(23) = −2.73, *p* = .01, *d* = -.56. Overall alexithymia symptoms did not significantly change over time, as indicated by the TAS-26 total score, *F*(1.36, 31.22) = 2.76, *p* = .10, eta^2^ = .10 (see Fig. 5D)). The subscales ‘Difficulty describing feelings’ and ‘External thinking’ did not significantly change, whereas ‘Identification of one's feelings’ did significantly change over time, *F*(2,46) = 6.25, *p* < .01, eta^2^ = .21 (see Table 1). No significant main effect of time was found for the total score of the ALS and the DSS-21 (see Table 1).

Original data without imputation revealed a nonsignificant main effect of time for the SEK total score, *F*(1.59, 27.07) = 3.34, *p* = .06, eta^2^ = .16, but a significant main effect of time for the TAS total score, *F*(2,36) = 5.33, *p* = .01, eta^2^ = .27. Post hoc paired *t*-test of original TAS total scores revealed a significant reduction from T1 to T2, *t*(18) = 2.78, *p* = .01 and from T1 to T2, *t*(18) = 2.56, *p* = .02. Results of the original TAS subscales can be found in the supplement.

To follow a conservative approach, we further discuss and interpret the original instead of the imputed data in case they differ from the imputed data. A detailed perspective of interviewer- and self-assessment results at T0, T1, and T2 can be found in Table 1.

### 3.3 Everyday experience: EMA

**Fig. 6:**
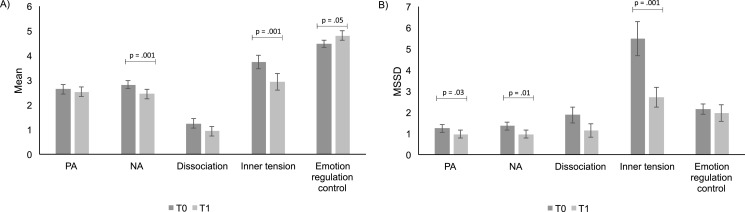
Ecological Momentary Assessment (EMA) data were assessed before (T0) and after (T1) neurofeedback training. A) Mean negative affect (NA) and inner tension significantly decreased, and perceived control over one's own emotions increased from T0–T1. Perceived control over one's own emotions was assessed with two items: asking how much participants felt they can control / cannot control their emotions now (see supplement for exact wording). Mean dissociation and positive affect (PA) did not significantly change from T0–T1. B) Mean squared successive differences (MSSD; i.e., hour-to-hour variability) of PA, NA, and inner tension significantly decreased from T0–T1, while the hour-to-hour variability of dissociation and perceived control over one's own emotions did not significantly change from T0–T1. Error bars represent standard error of the mean (SEM).

Explorative paired *t*-tests contrasting T0 and T1 revealed a significant reduction of mean negative affect (NA), *t*(23) = 3.46, *p* < .01, *d* = .70, a significant reduction of mean inner tension, *t*(23) = 3.27, *p* < .01, *d* = .67, a nonsignificant reduction of mean dissociative symptoms, *t*(23) = 1.85, *p* = .08, *d* = .38, and a significant increase of mean emotion regulation control, *t*(23) = −2.07, *p* = .05, *d* = -.42 (see Fig. 6A). No significant effects were found for mean positive affect (PA), *t*(23) = 1.28, *p* = .21, *d* = .26. Paired *t*-tests of the MSSDs revealed a significant reduction of instability in PA, *t*(23) = 2.30, *p* = .03, *d* = .47, NA, *t*(23) = 2.73, *p* = .01, *d* = .56, and inner tension, *t*(23) = 3.41, *p* < .01, *d* = .18 (see Fig. 6B). No significant effects were found for the instability of dissociative symptomatology, *t*(23) = 1.71, *p* = .10, *d* = .35. Adherence to prompts was 69.21% (SD = 18.18) at T0 and 63.87% (SD = 17.34) at T1, which is satisfactory. There was no significant difference in adherence between T0 and T1, *t*(23) = .16, *p* = .12.

## 3.4 Behavior: emotion regulation test and resting HRV

**Fig. 7:**
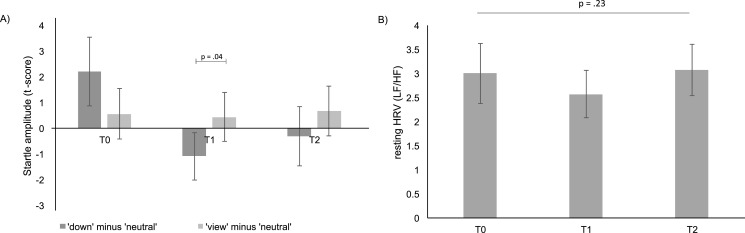
A) Mean startle amplitudes in the ‘down’ and ‘view’ condition at each assessment (T0, T1, and T2). Mean amplitudes represent the *T-*score converted to difference scores (‘down’ minus ‘neutral’ and ‘view’ minus ‘neutral’). Results indicate a significant reduction of emotion-modulated startle amplitude in the ‘down’ versus ‘view’ condition at T1, but not at T0 or T2. B) Resting HRV did not significantly change over the course of the study. Error bars represent standard error of the mean (SEM).

As hypothesized, patients could downregulate negative emotions more effectively after training, indexed by a significant decrease of the emotion-modulated startle in the ‘down’ compared to the ‘view’ condition after training, *F*(2,46) = 4.23, *p* = .02, eta^2^ = .16 (uncorrected^2^). There was no significant main effect of time, *F*(2,46) = .90, *p* = .42 and condition, *F*(1,23) = .39, *p* = .54. The interaction was due to a significant difference between the ‘down’-‘neutral’ and the ‘view’-‘neutral’ condition at T1, *t*(23) = −2.15, *p* = .04, *d* = -.44. In T0 and T2, in contrast, patients did not significantly decrease startle in the ‘down’-‘neutral’ vs ‘view’- ‘neutral’ comparison (see Fig. 7A).

Results from original data revealed similar results, except for the post hoc paired *t*-tests of original emotion-modulated startle data: Emotion-modulated startle was lower in the ‘down’ than the ‘view’ condition at T1, but this effect was only at the trend-level, *t*(17) = −2.01, *p* = .06, *d* = -.47.

Arousal ratings of the emotion regulation test (‘down’-‘neutral’; ‘view’-‘neutral’ significantly changed over time, corroborated by a significant main effect of time, *F* (2,46) = 18.64, *p* < .01, eta^2^ = .51. A significant main effect of condition indicated that overall arousal ratings were significantly lower in the ‘down’-‘neutral’ than in the ‘view’-‘neutral’ condition, *F*(1,23) = 3.33, *p* = .03, eta^2^ = .23. Post hoc *t*-tests between the ‘down’ and ‘view’ condition at T0, T1, and T2, respectively, revealed no significant effects, all *p*s > .10. Valence ratings of the emotion regulation test (‘down’-‘neutral;’ ‘view’-‘neutral’) substantially changed over time, corroborated by the main effect of time, *F* (2,46) = 3.22, *p* = .05, eta^2^ = .16. Interaction of time and condition was not significant.

Resting HRV did not change over time, *F*(1.33, 23.870) = 1.27, *p* = .23, eta^2^ = .07 (see Fig. 7B).

### 3.5 Brain responses: EWMT and BMT

**Fig. 8:**
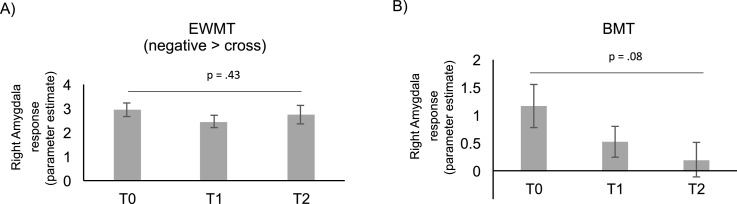
A) Right amygdala hemodynamic response during the Emotional Working Memory Task (EWMT) for each visit (T0, T1, T2). Amygdala hemodynamic response was assessed using fMRI during exposure to negative pictures, versus pictures depicting a fixation cross (negative > cross). B) Right amygdala hemodynamic response during the Backward Masking Task (BMT) for each visit (T0, T1, and T2). Amygdala hemodynamic response was assessed using fMRI during exposure to fearful and happy faces. Error bars indicate standard error of the mean (SEM).

EWMT accuracy was not significantly different between conditions and did not improve, all *p*s > .10. EWMT reaction times were significantly increased in the ‘negative’ versus ‘cross’ condition, *F*(2,46) = 3.66, *p* = .03, eta^2^ = .14, but did not change over time, as corroborated by a nonsignificant interaction of time and condition, *p* > .10. Contrary to our hypothesis, amygdala reactivity did not change over EWMT sessions, indicated by a nonsignificant interaction effect, *F*(2,46) = .87, *p* = .43. eta^2^ = .04 (see Fig. 8A).

Regarding the BMT, amygdala activity to quickly-presented happy and fearful faces did not significantly change after neurofeedback, although a decreasing trend could be observed, *F* (2,46) = 2.74, *p* = .08, eta^2^ = 0.11 (see Fig. 8B).

### 3.6 Correction for multiple testing

We repeated analyses on primary endpoints (ZAN-BP and DERS total score, emotion-modulated startle, resting HRV, amygdala reactivity to EWMT and BMT) using a Bonferroni-corrected alpha-level of *p* = .008. None of the main effects remain statistically significant with Bonferroni-correction.

### 3.7 Correlations between neurofeedback success and outcome measures

There were no significant correlations between amygdala down-regulation at run 4 and any of the primary endpoints (see supplement Table S3). Amygdala amplitude down-regulation at run 1 correlated significantly and positively with resting HRV at T0 (*r* = .51, *p* = .01, *N* = 24; not significant with Bonferroni-correction), and significantly and negatively with the ZAN-BPD total score at T0 (*r* = -.45, *p* = .03, *N* = 24; not significant with Bonferroni-correction). Changes in downregulation of amygdala amplitude and AUC during neurofeedback and changes in primary endpoints did not significantly correlate (see supplement Table S3 for results). Similarly, there were no significant correlations between participants’ best performance and changes in measures with a-priori hypotheses (Table S4).

## 4 Discussion

This is the first study assessing alterations in a variety of emotion processing and emotion regulation measures after amygdala neurofeedback training for BPD. This is an important step towards advancing neurobiological models and treatment for BPD, using endogenous neuromodulation with neurofeedback. Our results show that BPD patients were able to downregulate amygdala activation with neurofeedback. BPD psychopathology, emotion dysregulation, and affective instability improved at several levels of analysis, including self-report, startle modulation, and experience in everyday life. With regard to our primary endpoints, effects failed to pass significance level when applying a conservative correction for multiple tests. Therefore, our results need to be treated as preliminary and should be replicated by an independent study.

In line with our hypotheses, we observed changes in the ZAN-BPD interview, suggesting that subjectively-experienced BPD symptoms improved over the course of the study. These results are in accordance with other studies reporting associations of amygdala normalization and reductions in BPD psychopathology, and are in harmony with the notion that amygdala response is a critical mechanism of remission with BPD (Goodman et al., 2014; Schnell and Herpertz, 2007).

In addition, the present results on our EMA analyses indicate that negative affect and affective instability experienced in daily life reduced over time as well. Affective instability in BPD is supposed to arise from high sensitivity of neural systems involved in the generation of an emotional state, in combination with a severe emotion regulation deficit (Koenigsberg, 2010; Putnam and Silk, 2005). Increased amygdala activation has been interpreted as impairment in top-down control of the prefrontal cortex and may therefore contribute to affective instability (Dillon and Pizzagalli, 2007; Herpertz et al., 2018; Schulze et al., 2016). Amygdala neurofeedback training might be specifically suited to target the neural mechanisms of affective instability in precision psychiatry, although more research is needed for corroboration.

On the physiological level, we found an improvement in emotion regulation after training, evidenced by reduced startle-response, which suggests that participants improved their ability to regulate negative emotions. The neural pathway of the emotion-modulated startle involves midbrain neurons, mainly controlled by the central nucleus of the amygdala (Rosen et al., 1991). Enhanced amygdala activation leads to enhanced startle response (Davis et al., 1999; Rosen and Davis, 1988). Given the strong relation between emotional dysregulation, enhanced amygdala activation, and enhanced startle response (Davis et al., 1999; Rosen and Davis, 1988), our results suggest that emotion-modulated startle is a sensitive measure for investigating therapeutic effects of amygdala neuromodulation. Improvements in emotion regulation, assessed with the emotion-modulated startle, however, faded to the follow-up test; that is, some training effects did not persist for 6 weeks. Future studies must gain more stable effects, such as adding booster sessions or homework between sessions (Paret et al., 2019).

Contrary to our hypotheses, we did not find significant changes at the brain level. That is, amygdala response to negative pictures and facial expressions did not significantly lessen after neurofeedback training. In addition, no significant changes in resting HRV were observed. A possible explanation could be that these tasks simply do not measure the mechanisms that are trained with neurofeedback. During the EWMT and BMT, participants viewed emotional pictures, but were not explicitly told to regulate their emotions. Rather, these tasks measure the spontaneous response to negative stimuli. Likewise, resting HRV is a measure of autonomic flexibility representing the capacity for spontaneously regulated emotional responses (Appelhans and Luecken, 2006). In contrast, participants showed improvements in the emotion regulation test after training. The emotion regulation test explicitly instructed participants to downregulate negative emotions. In other words, our treatment might not alter the spontaneous response to negative emotions. Rather, participants might have acquired new or already-strengthened existing emotion regulation skills.

With respect to alexithymia, i.e. the difficulty to cognitively process emotions, our results suggest a reduction in these symptoms after training. However, we highlight the explorative fashion of this finding and we stress that only the original data showed significant reductions. Nonetheless, our results are in line with a recent study showing that amygdala electrical fingerprint neurofeedback resulted in a larger reduction of alexithymia scores compared to a control group (Keynan et al., 2019). Conversely, neurofeedback studies to increase the amygdala response showed that the ability to identify or describe one's own emotions (as indicated by a subscale of the Toronto alexithymia scale; TAS), was correlated with the effectivity to increase amygdala activity (Young et al., 2017, 2014; Zotev et al., 2011), which suggests that individuals with less symptoms of alexithymia might have better prerequisites to learn increasing their amygdala activity with neurofeedback. Together with our results, these studies indicate that the ability to identify and describe one's own feelings is directly related to the ability to gain control over the amygdala, however further studies are needed to fully understand the relation between alexithymia and amygdala neurofeedback.

Overall, patients were able to downregulate the amygdala BOLD response with feedback, which is in line with our prior study (Paret et al., 2016). However, when looking at each run individually, we could not observe a significant downregulation effect in all four runs. Rather, the difference between the ‘down’ and ‘view’ condition descriptively seemed to increase over time (although the interaction of run and condition did not pass the significance level). In particular, significant downregulation of the amygdala amplitude was achieved at the fourth training run and downregulation of the AUC was achieved at the second and fourth run. This implies that in BPD patients multiple training runs are necessary to observe amygdala downregulation with neurofeedback.

In addition, we determined participants’ best performance (i.e. the run with the largest delta between ‘view’ and ‘down’). Both downregulation of the amygdala BOLD response and best performance did not correlate significantly with any of our primary endpoints. Thus, evidence for a mechanistic relationship between amygdala regulation and emotion dysregulation is still missing. The lack of significance may be a function of several causes, including lack of power and technical issues. For example, the neurofeedback training was optimized to increase absolute training time but was less optimal in terms of quantifying downregulation of the amygdala, as the view condition of each session was comprised of only five pictures, while the ‘down’ condition was comprised of 25 pictures. Additionally, shifts of behavior, physiology, and cognition during an emotional response are often loosely coupled (Bonanno and Keltner, 2004), and as such, a significant correlation is not necessarily observable, particularly in small sample sizes. Placebo-controlled trials are necessary to corroborate that neurofeedback training is indeed causal for improvement in emotion regulation.

### 4.1 Limitations

Several limitations merit comment. Most importantly, the present study lacks a control group, so that our results do not allow conclusions about the specificity and efficacy of neurofeedback training. It is possible that factors other than the neurofeedback training itself account for the results. For example, it could be that the motivation to try a new treatment approach, psychosocial factors or effects of repeated exposure of tasks (i.e. practice effects) led to the observed changes. Therefore, replication in a randomized controlled trial is necessary. In addition, we assessed a large number of different outcome measures. Testing many different outcome measures in a single patient cohort is the only way to identify potential behavioral targets for a new, technically-demanding, and cost-intensive technique (such as neurofeedback), given the current database and limited financial resources. Multiple comparisons however bare the risk of false discovery. To overcome this issue, we repeated statistical tests of primary endpoints with a conservative correction for multiple tests (i.e. Bonferroni-correction). No statistical tests survived significance testing with correction. Notwithstanding such disenchanting outcome, several comparisons (e.g. ZAN-BPD, startle response) achieved medium effect sizes. With appropriate sample size, future studies might replicate this finding and achieve significant outcomes.

Finally, the fixed order of the EWMT and BMT in the experiment might induce bias. Both tasks were performed prior to the first scanning session and immediately after the last neurofeedback training. At the end of the last scanning session, participants might have been fatigued and less capable or motivated to concentrate. Similarly, results from the ZAN-BPD should be interpreted with caution, as EMA assessment was conducted one week before the ZAN-BPD interview and may have biased the effect, as interviewers were not blinded to treatment.

### 4.2 Conclusions

Until now, it has been unclear which aspects of psychopathology and emotion regulation may change with neurofeedback-aided amygdala downregulation. The present study provides the first preliminary empirical basis for informed decision-making in primary outcome measures of larger clinical trials of amygdala neurofeedback training. We show that general BPD psychopathology, as well as different aspects of emotion dysregulation, improve after training, although these effects do not remain statistically significant after a conservative correction for multiple tests. If confirmed by an independent study, our results suggest that the ZAN-BPD, emotion regulation (assessed with emotion-modulated startle), and EMA are appropriate measures to quantify these improvements. Future placebo-controlled trials must confirm that neurofeedback treatment is effective in improving emotion regulation in those with BPD. Future trials will allow for the development of new therapy concepts, including neurofeedback that can be incorporated into clinical practice. In addition, the causal pathway through amygdala hyperactivation, regarding symptoms of emotion dysregulation, can also be tested.

## Appendix Supplementary materials

Image, application 1

## Footnotes

1 104 dB startle probes elicited severe discomfort in two participants. Therefore, we turned down the volume to 95 dB for them. We reran startle analyses without the two participants, without the results changing. Thus, we report data from all participants.

2 no correction for multiple testing was applied.
