# Supplementary material for: Improved emotion regulation after neurofeedback: A single-arm trial in patients with borderline personality disorder
Source: Neuroimage Clin. 2019 Oct 16;24:102032. doi: 10.1016/j.nicl.2019.102032 (PMC6978219; doi:10.1016/j.nicl.2019.102032)
Supplement: Supplementary file 1 [file mmc1.docx]

**Supplement**

**Improved emotion regulation after neurofeedback:**

**A single-arm trial in patients with borderline personality disorder**

Jenny Zaehringer*^1^, Gabriele Ende^2^, Philip Santangelo^3^, Nikolaus Kleindienst^4^, Matthias Ruf^2^, Katja Bertsch^5^, Martin Bohus^4^, Christian Schmahl^1^ & Christian Paret*^1,6^

^1^ Department of Psychosomatic Medicine and Psychotherapy, Central Institute of Mental Health, Medical Faculty Mannheim / University of Heidelberg, Mannheim, Germany

^2^ Department Neuroimaging, Central Institute of Mental Health, Medical Faculty Mannheim / University of Heidelberg, Mannheim, Germany

^3^ Chair of Applied Psychology / Mental Health Lab, Institute of Sport and Sports Science, Karlsruhe Institute of Technology, Karlsruhe, Germany

^4^ Institute of Psychiatric and Psychosomatic Psychotherapy, Central Institute of Mental Health, Medical Faculty Mannheim / University of Heidelberg, Mannheim, Germany

^5^ Department for General Psychiatry, Center of Psychosocial Medicine, University of Heidelberg, Heidelberg, Germany

^6^ Sagol Brain Institute, Wohl Institute for Advanced Imaging, Tel-Aviv Sourasky Medical Center and School of Psychological Sciences, Tel-Aviv University, Tel-Aviv, Israel

* Correspondence to

Jenny Zaehringer

Central Institute of Mental Health

J5, D-68159 Mannheim, Germany

Email: jenny.zaehringer@zi-mannheim.de

Tel: +49-(0)-621-1703-4402

Christian Paret

Central Institute of Mental Health

J5, D-68159 Mannheim, Germany

Email: christian.paret@zi-mannheim.de

Tel: +49-(0)-621-1703-4462

1. **Supplementary Methods**
   1. **Flow chart of the study**

## Enrollment

**Assessed for eligibility (n=108)**

**Excluded (n=77)**

- Did not meet inclusion criteria (n = 50)
- Not interested in the first place (n = 19)
- Ultimately did not participate (n = 8)

## Allocation

**Allocated to intervention (n=31)**

- Received allocated intervention (n=26)
- Did not receive allocated intervention (technical problems) (n=2)
- Discontinued intervention (unexpected hospital stay during intervention, too much time between NFB sessions) (n=3)

## Follow-Up

**Completed follow-up (n=24**)

- Lost to follow-up (didn’t attend the follow-up session) (n=2)

## Analysis

**Analyzed (n=22)**

- Excluded from analysis (amphetamine consumption during treatment, slept during neurofeedback sessions) (n=2)

*Figure 1.* CONSORT flow chart for the study.

- 1. **Recruitment**

Recruitment was done by the central project of the KFO-256, a Clinical Research Unit funded by the German Research Foundation (DFG; KFO-256). Interested patients were told about the aims of the study as follows:

“fMRI neurofeedback is an innovative technique with which brain regulation can be improved. Using fMRI, real-time brain activation is measured and fed back to the patient in real-time. In this vein, self-regulation and emotion processing may be improved. In this study we would like to investigate the therapeutic effects of this technique for borderline personality disorder.”

- 1. **Real-time fMRI neurofeedback**
     1. **Procedure**

BPD patients participated in a total of three neurofeedback training days with an interval of 2–7 days between subsequent sessions. On the second training day, participants underwent two consecutive training runs, whereas on the first and third training day they underwent one training run. A neurofeedback training run lasted 15 mins.

Before the neurofeedback training started, participants were told that they would see negative pictures and a feedback signal from their brain depicted as a colored thermometer bar on each side of the picture. They were further instructed to down-regulate the thermometer bar and that there would be a temporal delay of the BOLD response, which caused a time lag of the thermometer response (2-5s). In addition, they were instructed to keep their eyes open, not shift their gaze away from the screen, not to focus exclusively on the thermometer and the edges of the picture, not to control their breath and to keep their heads still throughout the experiment. Participants then entered the scanner and the experiment started. After the anatomical scans were acquired, a demo-feedback trial was presented without fMRI scanning to get participants accustomed to the training. Subjects were instructed to either look at the picture (without receiving feedback), or to down-regulate the thermometer signal, respectively. The neurofeedback run thus consisted of ‘down’ and ‘view’ conditions. In the ‘down’ condition, the pictures were presented with feedback. In the ‘view’ condition, a picture with aversive content was shown. A neurofeedback run comprised ten blocks, with five ‘down’ blocks consisting of six pictures presented for 18s (108s total) and with five ‘view’ blocks consisting of one picture presented for 18s. The order of conditions was fixed with alternating view and down blocks. After the last block, participants were instructed to rate their perceived regulation success (‘Were you able to regulate the display?’) on a 10-level visual analogue scale.

- - 1. **Stimuli used for neurofeedback runs**

For the neurofeedback training runs, 132 negative stimuli were taken from standardized picture series (Dan-Glauser & Scherer, 2011; Lang, Bradley, & Cuthbert, 2008; Marchewka, Zurawski, Jednoróg, & Grabowska, 2014; Wessa et al., 2010) and were completed with 8 pictures from the internet. Stimuli were presented with the Presentation software (Neurobehavioral Systems, Berkeley, CA). For each patient, pictures were randomly assigned to runs.

Stimuli with these original codes were used:

EmoPics: 208, 210, 211, 212, 213, 214, 215, 216, 217, 218, 219, 220, 221, 222, 223, 224, 225, 226, 227, 228, 229, 230, 231, 232, 241, 243, 244, 245, 246, 251, 252, 253, 254.

IAPS: 2053, 2301, 2345, 2375, 2456, 2457, 2458, 2691, 2694, 2700, 2703, 2718, 2795, 2799, 2800, 2900, 2981, 3180, 3215, 3220, 3500, 3530, 6220, 6231, 6311, 6313, 6520, 6550, 6563, 6831, 6940, 8485, 9050, 9163, 9183, 9185, 9230, 9250, 9332, 9342, 9414, 9415, 9419, 9423, 9424, 9425, 9426, 9428, 9429, 9435, 9480, 9901, 9902, 9905, 9920.

EmoMadrid: EM0001, EM0123, EM0319, EM0321, EM0322, EM0326, EM0331, EM0350, EM0356, EM0363, EM0392, EM0393, EM0410, EM0420, EM0447, EM0484, EM0488, EM0568, EM0570, EM0572, EM0577, EM0586, EM0597, EM0605, EM0633, EM0692

NAPS: Faces_012_v, Faces_021_h, Faces_148_h, Faces_150_h, Faces_303_h, Landscapes_002_h, Objects_006, Objects_011_h, Objects_012_h, Objects_125_h, Objects_143_h, Objects_275_v, People_071_h, People_079_h, People_118_h, People_139_h, People_210_h, People_243_h.

- - 1. **Neurofeedback strategies used**

After each neurofeedback session, participants indicated which strategies they used to down-regulate the feedback signal and to what extent. We used an inhouse questionnaire that was created based on our experience of previous neurofeedback studies. In the questionnaire, participants were provided with different cognitive strategies such as reappraisal, distraction and mindfulness strategies and were instructed to indicate on a visual analogue scale to what extent they have used the strategy during the neurofeedback session.

The following strategies were provided:

1. *I tried to reappraise the depicted situation (e.g. I told myself that this is just a film).*
2. *I tried to distance myself from the depicted situation (e.g. I told myself that this doesn't pertain to me).*
3. *I tried to distract myself with different thoughts or activities (e.g. mental arithmetic, counting numbers).*
4. *I tried to relax (e.g. by focusing on my breath or by doing relaxation exercises).*
5. *I tried to look at the picture without evaluating it (e.g. by describing what I see on the picture without evaluating it in a positive or negative manner).*
6. *I tried to shift my attention away from aversive details of the picture and to e.g. relocate my attention towards neutral details of the picture or towards the thermometer display.*
7. *I tried to remember positive emotional memories e.g. with my family or friends.*
8. *I tried to think of a positive story of the people depicted on the picture (e.g. the person will get help, or the person is doing better).*
9. *I tried to think of a reason or a story behind the depicted situation.*
10. *I tried to integrate the picture into a political or historical context.*

Participants were instructed to mark on a continuous analogue scale, how often they used the strategies provided. The manually set marks were digitally transformed into a percent maximum possible ranging from 0 to 100. Later, question 1, 2, 8, 9 and 10 were combined to *reappraisal* strategies, question 3 and 7 were combined to *distraction* strategies, by adding the mean values of the respective strategies and subdividing it by the number of strategies. Thus, we analyzed 5 strategies: *reappraisal, distraction, relaxation, mindful viewing, paying attention to neutral aspects*.

- - 1. **Real-time fMRI analysis and feedback presentation**

Scan volumes were immediately transferred from the scanner site to a computer in order to preprocess and analyze the data using SPM8 (Wellcome Department of Cognitive Neurology, London, UK). For that, the T1-weighted scan was first segmented and normalized to Montreal Neurological Imaging (MNI) space. Anatomical masks of the regions of interest (ROI) for feedback calculation were then moved to subject-native space. The BOLD signal data from voxels within a right amygdala mask was taken for further processing, The mask was created with the Harvard-Oxford brain atlas with a probability threshold of 25%. We also recorded BOLD signal data from a rectangular ROI (3x30x30 mm in AC-PC orientation, center of mass = [0,-16,-5], MNI coordinates). This mask served as a control for global signal fluctuation unrelated to functional brain activation. The perpendicular distance from the control ROI to the right amygdala mask comprised 7 mm in sagittal direction. Functional images were realigned to the first volume and BOLD signal data from all voxels within each ROI were averaged. The average timecourse was processed with a modified Kalman filter (Koush, Zvyagintsev, Dyck, Mathiak, & Mathiak, 2012) and detrended using Matlab’s (R2014b) detrend function. Detrending began with the 35th volume (i.e. before feedback started) so that the filter and detrend functions were stabilized. Next, percent signal change from the global mean was calculated. On a second computer, stimulus presentation software received the data via TCP/IP. The feedback was displayed as a colored rectangle moving up and down, altering from dark red at the maximum over light green to dark green at the minimum. Resolution of the display consisted of six units and ranged between two percent signal change above and below baseline. The display was refreshed with a frequency of 1 Hz. Details of fMRI acquisition, real-time fMRI analysis and feedback presentation can be found in the supplement and has been published by Paret et al. (2018).

- 1. **Emotion regulation test**
     1. **Procedure**

Participants completed the task directly before (T0) and after (T1) the neurofeedback training as well as after 6 weeks (T2). They were instructed either to view negative and neutral pictures without modifying their emotions (‘view’; ‘neutral’ condition respectively) or to down-regulate their feelings toward negative pictures (‘down’ condition). Furthermore, participants were instructed not to turn away their gaze or to close their eyes, nor to focus exclusively on non-emotional parts of the picture. Before the emotion regulation task started, ten startle probes were presented consecutively, to control for habituation effects. In total, the paradigm consisted of 36 trials (12 trials per condition) and lasted 20 min. Each trial began with a 2,000-ms presentation of an instructional cue (‘view’, ‘down’), followed by a fixation cross displayed for 1,000 ms. Next, a neutral or negative picture was presented for 10,000 ms. A startle probe (50 ms, 95dB white noise burst) was presented through headphones at 6,500 ms – 9,500 ms into the regulation phase). Self-assessment Manikins (SAM Ratings;(Bradley & Lang, 1994)) were presented after presentation of each picture. Participants rated on a 1-9 Likert scale how positive/negative and aroused/calm they felt at that moment. Lower scores on the valence scale indicate that they felt more positive; lower scores on the arousal scale indicate that they felt calmer. By pressing buttons on a keyboard, subjects selected SAMs corresponding to their subjective valence and arousal. The initial rating position was random and the current selection after 5 s was logged. Intertrial intervals were jittered between 3,500 and 5,500 ms. Picture stimuli were presented in semi-randomized order with restriction of no more than two consecutive trials from the same condition, and no more than three consecutive trials with negative pictures. The participants’ eyes were tracked by a camera system (SMI BeGaze, Teltow, Germany) to encourage subjects to comply with instructions, data were not analyzed. Ten percent of trials did not include a startle probe (2 trials per condition).

- - 1. **Stimuli used for the emotion regulation test**

Stimuli were taken from standardized picture series (Dan-Glauser & Scherer, 2011; Lang, Bradley, & Cuthbert, 2008; Marchewka, Zurawski, Jednoróg, & Grabowska, 2014; Wessa et al., 2010) and were presented with the Presentation software (Neurobehavioral Systems, Berkeley, CA). Arousal and valence were calculated from normative ratings provided with the published data sets. Pictures were matched between T-assessments according to arousal and valence. Stimuli with the these original codes were used:

T0: Negative: 247, 1033, 1304, 2205, 3185, 6212, 6510, 9413, 9433, 9921, People_034, Animals_033, Animals_052, People_147, Faces_003, Faces_028, Faces_041, Faces_272, People_002, People_007, People_039, People_072, People_136, People_203. Neutral: 2381, 5510, 5740, 7003, 7025, 7052, 7150, 7161, 7175, 7185, 7236, 7950.

T1: Negative: 234, 239, 250, 252, 3181, 6244, 9042, 9412, Animals_001, Animals_014, Animals_034, Faces_014, Faces_146, Faces_174, Faces_274, Faces_290, Faces_369, Objects_139, Objects_283, People_019, People_084, People_124, People_143, Animals_076- Neutral: 2038, 6150, 7000, 7004, 7006, 7010, 7035, 7041, 7045, 7187, 7205, 7490.

T2: Negative: 209, 249, 2683, 235, 325, 6231, 9904, 2811, Animals_003, Animals_006, Faces_035, People_145, Faces_151, People_033, People_231, Animals_085, People_004, People_073, Faces_007, Faces_034, Faces_019, People_001, Faces_293, People_038. Neutral: 7001, 7026, 7217, 7080, 5471, 2580, 7090, 7224, 5635, 7100, 7009, 7002.

- 1. **Emotional Working Memory Task (EWMT)**
     1. **Procedure**

The EWMT is an adapted Sternberg item recognition task (Sternberg, 1966), modified by Oei and colleagues (Krause-Utz et al., 2014; Krause-Utz et al., 2012; Oei et al., 2012). The present version comprised 40 trials with jittered durations: each trial started with the presentation of a set of three letters (memoranda, 875 – 1,375 ms). After a delay phase of 1,250-1,750 ms another set of three letters appeared on the screen (probe, 2,000ms). Next, a blank screen appeared (inter trial interval, 550 – 1,050 ms). Participants had to press the left or right button to indicate whether they recognized one of the memoranda-letters in the probe. In half of the trials, one of the three memoranda was present in the probe. During the delay interval, either no distractor (i.e. a fixation cross; ‘cross’ condition) or a distractor (i.e. an aversive picture; ’negative’ condition) was presented. Target-present and target-absent trials were equal across the ‘negative’ and the ‘cross’ condition. The presentation of the two conditions (write conditions) was balanced in a pseudo-random manner with no more than two consecutive conditions of the same type. Stimuli were presented in semi-randomized order with restriction of no more than two consecutive trials from the same condition (write conditions), and no more than three consecutive trials with negative pictures. Order of condition and assignment of pictures to assessments (i.e. T0, T1, T2) was alternated between subjects.

- - 1. **Stimuli used for the EWMT**

Stimuli were taken from standardized picture series (Dan-Glauser & Scherer, 2011; Lang, Bradley, & Cuthbert, 2008; Marchewka, Zurawski, Jednoróg, & Grabowska, 2014; Wessa et al., 2010) and were presented with the Presentation software (Neurobehavioral Systems, Berkeley, CA). Pictures were matched between T-assessments according to arousal and valence calculated from normative ratings provided in original publication of the data sets. The following stimuli were used:

T0: 284, 242, 1202, 3550, 6021, 9620, 3195, 9810, 6370, 1300, 9635, 1202, 3212, 6350. People_022_h, Faces_363_h, People_209_h, Faces_284_h, People_031_h, People_128_h.

T1: 233, 3300, 8230, 9800, 9254, 6570, 9670, 9325, 3103, 9600, 9321, 2730, 3030, 9410, People_214, People_058, People_140, People_088, Objects_001, People_246.

T2: 237, 3350, 9181, 9300, 9400, 9910, 6415, 9075, 6834, 1930, 6312, 9252, 1120, 6230, Animals_008, People_225, People_037, Faces_362, Objects_149, Faces_010.

- 1. **Backward Masking Task (BMT)**
     1. **Procedure**

Participants were instructed to identify whether faces expressing either happy or fearful facial expressions are male or female. To keep them attentive throughout the task, participants were told to identify whether the presented faces were male or female via button press. Faces were presented for 33 ms or 83 ms. Thus, the BMT had a total of four conditions: Happy or fearful facial expressions either presented for 33 ms or 83 ms. A total of 4 blocks per condition were presented. Each block consisted of 8 pictures shown consecutively. Each block began with a fixation cross. Next, eight faces were shown for 33ms or 83ms, each preceded by a red rectangle on a grey background for 5,100 ms and followed by a mask (scrambled face) for 4,100 ms. Stimuli were taken from the Karolinska Directed Emotional Faces set (KDEF; (Lundqvist, Flykt, & Öhman, 1998)).

- - 1. **Behavioral data of the BMT**

Participants were instructed to identify the sex of the faces to keep them attentive throughout the task by pressing either the left or right button, respectively. Button presses were analyzed by summing up right, wrong and missed button presses per session across all conditions.

- 1. **EMA**
     1. **Questions**

**The following questions were used for EMA assessments:**

*Positive affect:*

At the moment I feel happy

At the moment I feel happy relaxed

At the moment I feel happy content

At the moment I feel happy enthusiastic

*Negative affect:*

At the moment I feel happy sad

At the moment I feel happy irritated

At the moment I feel happy angry

At the moment I feel happy depressed

At the moment I feel happy fearful

*Subjective control over emotions:*

When the phone rang I felt like I could control my feeling

When the phone rang I felt overwhelmed by my feelings

*Inner tension:*

At the moment I feel happy an aversive inner tension

*Dissociation:*

When the phone rang I felt like my body did not belong to me

When the phone rang I felt like people or things or the world were not real

When the phone rang I had problems to hear right, e.g. noise around me appeared as if it came from far away

When the phone rang I felt like my body or some body parts were insensitive to pain

**1.7 Consensus on the Reporting and Experimental Design of clinical and cognitive-behavioural Neurofeedback studies (CRED-nf) best practices checklist 2019**

| **Domain** | **Item #** | **Checklist item** | **Reported on page #** |
| --- | --- | --- | --- |
| **Pre-experiment** | | | |
|  | 1a | Pre-register experimental protocol and planned analyses | p. 6 |
|  | 1b | Justify sample size | In our opinion, N = 25 subjects were sufficient to reach our aims |
| **Control groups** | | | |
|  | 2a | Employ control group(s) or control condition(s) | Control condition: p.7, p.16; control group: p.2, p.29 |
|  | 2b | When leveraging experimental designs where a double-blind is possible, use a double-blind | No blinding possible |
|  | 2c | Blind those who rate the outcomes, and when possible, the statisticians involved | Not applicable |
|  | 2d | Examine to what extent participants and experimenters remain blinded | Not applicable |
|  | 2e | In clinical efficacy studies, employ a standard-of-care intervention group as a benchmark for improvement | Not applicable |
| **Control measures** | | | |
|  | 3a | Collect data on psychosocial factors | supplement p. 3 + p. 14 |
|  | 3b | Report whether participants were provided with a strategy | p. 7 |
|  | 3c | Report the strategies participants used | supplement p. 4 + p. 13 |
|  | 3d | Report methods used for online-data processing and artifact correction | p. 8, supplement p. 3 |
|  | 3e | Report condition and group effects for artifacts | - |
| **Feedback specifications** | | | |
|  | 4a | Report how the online-feature extraction was defined | p. 8 |
|  | 4b | Report and justify the reinforcement schedule | p. 8 |
|  | 4c | Report the feedback modality and content | p. 8 |
|  | 4d | Collect and report all brain activity variable(s) and/or contrasts used for feedback, as displayed to experimental participants | p. 15 |
|  | 4e | Report the hardware and software used | p. 6 + p. 8 |
| **Outcome measures** | | | |
| Brain | 5a | Report neurofeedback regulation success based on the feedback signal | - |
|  | 5b | Plot within-session and between-session regulation blocks of feedback variable(s), as well as pre-to-post resting baselines or contrasts | p. 19 |
|  | 5c | Statistically compare the experimental condition/group to the control condition(s)/group(s) (not only each group to baseline measures) | Not applicable |
| Behaviour | 6a | Include measures of clinical or behavioural significance, defined a priori, and describe whether they were reached | p. 89f + p. 20ff |
|  | 6b | Run correlational analyses between regulation success and behavioural outcomes | p. 18; p. 26 |
| **Data storage** | | |  |
|  | 7a | Upload all materials, analysis scripts, code, and raw data used for analyses, as well as final values, to an open access data repository, when feasible | - |

1. **Supplementary results**
   1. **Data without imputation**
      1. **Neurofeedback down-regulation**

Original data of Amygdala AUCs revealed a significant main effect of condition, F(1,18) = 7.57, p = .01, eta2 = .30, and non-significant main effect of time, F(3, 54) = 2.53, p = 0.08, eta2 = .12, but no significant interaction of time and condition, F(3,54) = .96, p = .42, eta2 = .05. Post-hoc paired t-tests of original data revealed a significant effect between regulate and view at session 4, t(18) = -2.52, p = .02. Original data of Amygdala amplitudes revealed a main effect of condition, F (1, 18) = 7.97, p = .01, eta2 = .31. There were no significant main effect of time and no significant interaction of time and condition. Post-hoc paired t-tests of original data revealed a significant effect between regulate and view at session 4, t(18) = -2.84, p = .01.

- - 1. **Neurofeedback down-regulation strategies**

Descriptively, participants used mindful viewing the most and distraction strategies the least across all sessions (see Figure S2).

*Figure S2.* Mean extent of use of different strategies during the neurofeedback sessions.

- - 1. **TAS-26**

Original data of the TAS-26 revealed a significant main effect of time of the TAS total score, F (1.47, 27.91) = 6.85, p = .01, eta2 = .27, and of the subscale ‘Identification of one’s feelings’, F (2, 40) = 6.63, p < .01, eta2 = .25. Post-hoc paired t-tests revealed a significant decrease of ‘Identification of one’s feelings’ from T1 to T2, t(20) = 2.06, p = .05, and from T1 to T2, t(20) = 3.05, p = .01, and a significant decrease of TAS total score from T1 to T2, t(18) = 2.78, p = .01, and from T0 to T2, t(18) = 2.55, p = .02). No significant main effect of time was found for the subscales‚ Difficulty describing feelings’, F(1.56, 29.64) = .67, p = .52, eta2 = .03), and ‘external thinking’, F (2, 38) = 2.59, p = .09, eta2 = .12.

- - 1. **Emotion regulation test**

As hypothesized, patients down-regulated negative emotions more effectively after training, indexed by a significant decrease of the emotion-modulated startle in the ‘down’ compared to the ‘view’ condition after training, F(2,34) = 3.27, p = .05, eta^2^ = .16. There was no significant main effect of time, F(2,34) = 1.09, p = .35, and condition, F(1,17) = .28, p = .60. Post-hoc paired t-tests between the ‘down’-‘neutral’ and the ‘view’-‘neutral’ condition revealed a trend-level effect at T1, t(17) = -2.01, p = .06, d = -.47. At T0 and T2, in contrast, patients did not significantly decrease startle in the ‘down’-‘neutral’ vs ‘view’- ‘neutral’.

- 1. **Supplementary results with imputation**
     1. **Best performance**


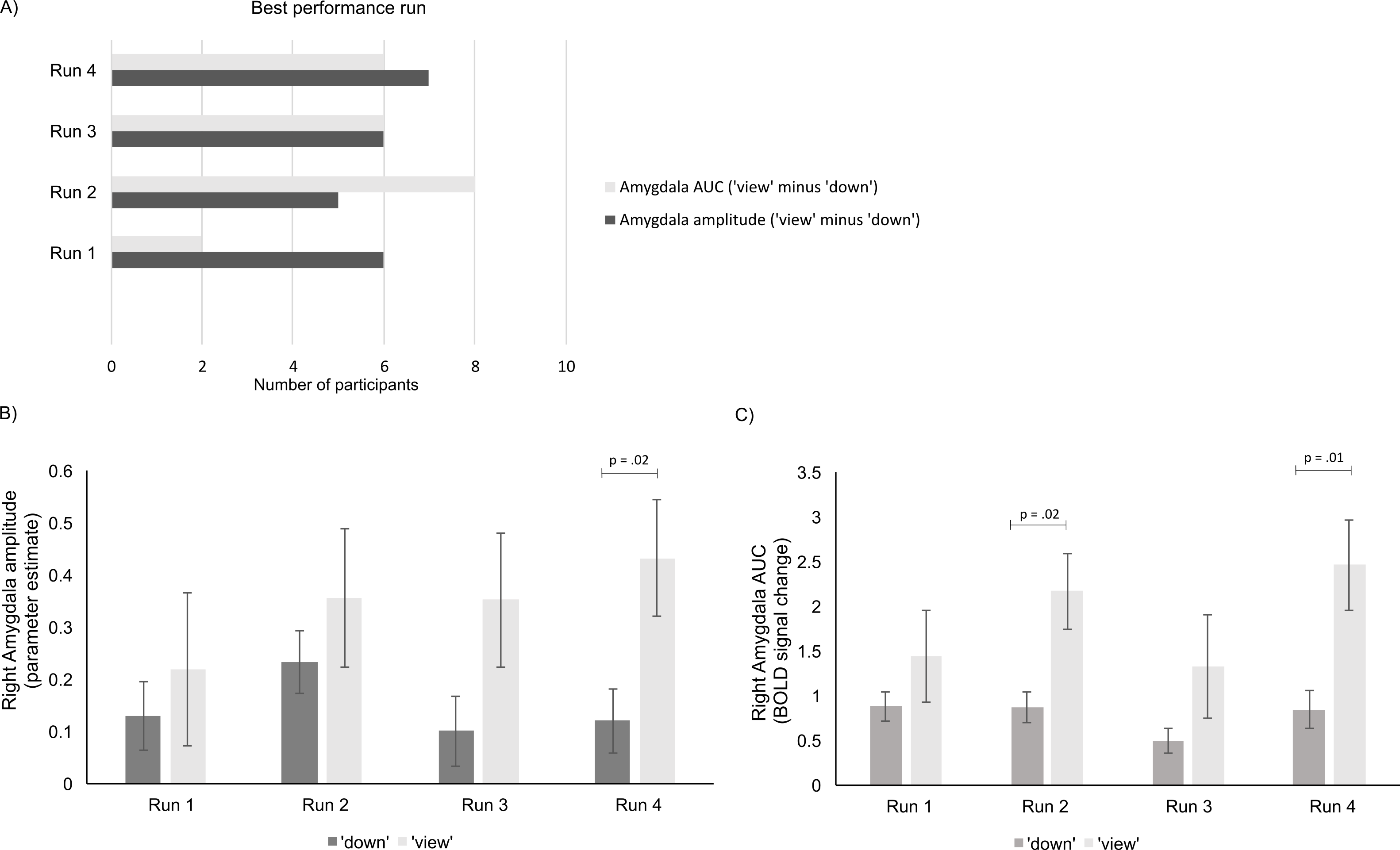
*Figure S3. Best performance (i.e. largest delta between ‘down’ and ‘view’) of amygdala downregulation. Bars represent for each run the number of participants that reached their best performance. AUC = area under the curve.*

- - 1. **Rating of subjective regulation success**

Participants were instructed to rate their perceived regulation success on a 10-level visual analogue scale (0 = not at all; 10 = very much). Mean success ratings ranged between 4.00 and 4.63 across sessions, suggesting that participants estimated their regulation success in the medium range. Success ratings did not significantly change over time, F(3,69) = 1.13, p = .34. Descriptive data of success ratings can be derived from Table S1.

Table S1. *Mean ratings of subjective down-regulation success during individual neurofeedback sessions*

|  | Session 1 | Session 2 | Session 3 | Session 4 |
| --- | --- | --- | --- | --- |
| Mean success (SD) | 4.00 (1.50) | 4.63 (1.86) | 4.56 (1.67) | 4.33 (1.34) |

- - 1. **Behavioral results of the BMT**

Mean number of right, F(2,46) = .64, p = .54, wrong, F(2,46) = .59, p = .56, and missed trials, F(2,46) = 1.33, p = .28, did not significantly change over time. Descriptive data can be derived from Table S2.

Table S2. *Mean (SD) of number of correct, wrong and missed trials during the BMT at T0, T1 and T2*

|  | Right answer | Wrong answer | Missed trials |
| --- | --- | --- | --- |
| T0 | 109.56 (14.87) | 14.51 (11.00) | 3.97 (5.45) |
| T1 | 110.49 (10.52) | 13.36 (8.51) | 4.10 (4.33) |
| T2 | 107.53 (22.39) | 13.31 (10.32) | 7.15(15.29) |

- - 1. **Correlations between downregulation success and primary endpoints**

| Table S3 *Correlations between Amygdala downregulation and primary endpoints* | | | | | | | | | | | | | |
| --- | --- | --- | --- | --- | --- | --- | --- | --- | --- | --- | --- | --- | --- |
|  | | Startle down-regulation T0 | Startle down-regulation T1 | EWMT T0 | EWMT T1 | BMT T0 | BMT T1 | Resting HRV T0 | Resting HRV T1 | ZAN-BPD total T0 | ZAN-BPD total T1 | DERS total T0 | DERS total T1 |
| Amygdala AUC session 1 | Pearson Correlation | 0.10 | -0.23 | -0.11 | 0.00 | 0.03 | 0.11 | 0.03 | -0.10 | -0.300 | -0.30 | -0.01 | 0.16 |
|  | Sig. (2-tailed) | 0.65 | 0.27 | 0.61 | 1.00 | 0.90 | 0.62 | 0.88 | 0.64 | 0.154 | 0.16 | 0.96 | 0.46 |
|  | N | 24 | 24 | 24 | 24 | 24 | 24 | 24 | 24 | 24 | 24 | 24 | 24 |
| Amygdala AUC session 4 | Pearson Correlation | 0.17 | 0.04 | -0.02 | 0.33 | -0.03 | 0.39 | 0.15 | -0.12 | 0.143 | 0.18 | -0.29 | -0.11 |
|  | Sig. (2-tailed) | 0.44 | 0.86 | 0.93 | 0.11 | 0.89 | 0.06 | 0.47 | 0.56 | 0.504 | 0.40 | 0.17 | 0.62 |
|  | N | 24 | 24 | 24 | 24 | 24 | 24 | 24 | 24 | 24 | 24 | 24 | 24 |
| Amygdala amplitude session 1 | Pearson Correlation | 0.01 | 0.01 | -0.01 | -0.06 | -0.05 | 0.02 | **,511^*^** | -0.07 | **-,446^*^** | -0.31 | -0.05 | 0.13 |
|  | Sig. (2-tailed) | 0.95 | 0.96 | 0.98 | 0.79 | 0.80 | 0.92 | **0.01** | 0.74 | **0.029** | 0.14 | 0.80 | 0.53 |
|  | N | 24 | 24 | 24 | 24 | 24 | 24 | **24** | 24 | **24** | 24 | 24 | 24 |
| Amygdala amplitude session 4 | Pearson Correlation | -0.02 | 0.13 | -0.21 | 0.35 | -0.14 | 0.29 | 0.21 | -0.13 | -0.005 | 0.17 | -0.33 | -0.16 |
|  | Sig. (2-tailed) | 0.93 | 0.54 | 0.32 | 0.09 | 0.52 | 0.18 | 0.32 | 0.53 | 0.983 | 0.43 | 0.12 | 0.45 |
|  | N | 24 | 24 | 24 | 24 | 24 | 24 | 24 | 24 | 24 | 24 | 24 | 24 |
| Note. AUC, area under the curve; EWMT, Emotional Working Memory Task; BMT, Backward Masking Task; HRV, heart rate variability;, difficulties with emotion regulation scale. | | | | | | | | | | | | | |
| **. Correlation is significant at the 0.01 level (2-tailed).  * Correlation is significant at the 0.05 level (2-tailed). | | | | | | | | | | | | | |

Table S4

*Correlations between downregulation success indices and changes in primary endpoints*

|  | | ZAN total score (T0 minus T1) | DERS total score (T0 minus T1) | Startle amplitude (T0 minus T1) | HRV (T0 minus T1) | BMT (T0 minus T1) | EWMT (T0 minus T1) |
| --- | --- | --- | --- | --- | --- | --- | --- |
| Best performance (AUC) | Pearson Correlation | 0.127 | -0.137 | -0.265 | 0.079 | 0.090 | -0.081 |
|  | Sig. (2-tailed) | 0.554 | 0.525 | 0.210 | 0.712 | 0.677 | 0.706 |
|  | N | 24 | 24 | 24 | 24 | 24 | 24 |
| Best performance (amplitude) | Pearson Correlation | -0.336 | 0.043 | -0.097 | -0.178 | 0.272 | 0.266 |
|  | Sig. (2-tailed) | 0.108 | 0.844 | 0.653 | 0.406 | 0.198 | 0.209 |
|  | N | 24 | 24 | 24 | 24 | 24 | 24 |
| AUC (run 1 minus run 4) | Pearson Correlation | -0.090 | -0.004 | 0.070 | -0.092 | 0.180 | 0.151 |
|  | Sig. (2-tailed) | 0.677 | 0.985 | 0.747 | 0.669 | 0.401 | 0.482 |
|  | N | 24 | 24 | 24 | 24 | 24 | 24 |
| Amplitude (run 1 minus run 4) | Pearson Correlation | -0.098 | -0.059 | 0.052 | 0.240 | 0.127 | 0.303 |
|  | Sig. (2-tailed) | 0.647 | 0.783 | 0.808 | 0.259 | 0.555 | 0.149 |
|  | N | 24 | 24 | 24 | 24 | 24 | 24 |
| Note. AUC, area under the curve; EWMT, Emotional Working Memory Task; BMT, Backward Masking Task; HRV, heart rate variability;, difficulties with emotion regulation scale.  **. Correlation is significant at the 0.01 level (2-tailed). | | | | | | | |
| *. Correlation is significant at the 0.05 level (2-tailed). | | | | | | | |

**References**

Bradley, M. M., & Lang, P. J. (1994). Measuring emotion: The Self-Assessment Manikin and the semantic differential. *Journal of Behavior Therapy and Experimental Psychiatry, 25*(1), 49-59. doi: 10.1016/0005-7916(94)90063-9

Dan-Glauser, E. S., & Scherer, K. R. (2011). The Geneva affective picture database (GAPED): a new 730-picture database focusing on valence and normative significance. *Behavior research methods, 43*(2), 468. doi:10.3758/s13428-011-0064-1.

Koush, Y., Zvyagintsev, M., Dyck, M., Mathiak, K. A., & Mathiak, K. (2012). Signal quality and Bayesian signal processing in neurofeedback based on real-time fMRI. *NeuroImage, 59*(1), 478-489. doi:10.1016/j.neuroimage.2011.07.076

Krause-Utz, A., Elzinga, B. M., Oei, N. Y., Paret, C., Niedtfeld, I., Spinhoven, P., . . . Schmahl, C. (2014). Amygdala and dorsal anterior cingulate connectivity during an emotional working memory task in borderline personality disorder patients with interpersonal trauma history. *Frontiers in human neuroscience, 8*. doi:10.3389/fnhum.2014.00848

Krause-Utz, A., Oei, N., Niedtfeld, I., Bohus, M., Spinhoven, P., Schmahl, C., & Elzinga, B. (2012). Influence of emotional distraction on working memory performance in borderline personality disorder. *Psychological medicine, 42*(10), 2181-2192. doi:10.1017/S0033291712000153

Lang, P. J., Bradley, M. M., & Cuthbert, B. N. (2008). International affective picture system (IAPS): Affective ratings of pictures and instruction manual. *Technical report A-8*.

Lundqvist, D., Flykt, A., & Öhman, A. (1998). The Karolinska directed emotional faces (KDEF). CD ROM from Department of Clinical Neuroscience, Psychology section, Karolinska Institutet, ISBN 91-630-7164-9.

Marchewka, A., Żurawski, Ł., Jednoróg, K., & Grabowska, A. (2014). The Nencki Affective Picture System (NAPS): Introduction to a novel, standardized, wide-range, high-quality, realistic picture database. *Behavior research methods, 46*(2), 596-610. doi:10.3758/s13428-013-0379-1

Oei, N. Y., Veer, I. M., Wolf, O. T., Spinhoven, P., Rombouts, S. A., & Elzinga, B. M. (2012). Stress shifts brain activation towards ventral ‘affective’areas during emotional distraction. *Social Cognitive and Affective Neuroscience, 7*(4), 403-412. doi:10.1093/scan/nsr024

Paret, C., Zaehringer, J., Ruf, M., Gerchen, M. F., Mall, S., Hendler, T., . . . Ende, G. (2018). Monitoring and control of amygdala neurofeedback involves distributed information processing in the human brain. *Human brain mapping*. doi:10.1002/hbm.24057

Wessa, M., Kanske, P., Neumeister, P., Bode, K., Heissler, J., & Schönfelder, S. (2010). EmoPics: Subjektive und psychophysiologische Evaluation neuen Bildmaterials für die klinisch-bio-psychologische Forschung. *Z Klin Psychol Psychother, 39*(Suppl. 1/11), 77.
